# Selecting a randomization method for a multi-center clinical trial with stochastic recruitment considerations

**DOI:** 10.1186/s12874-023-02131-z

**Published:** 2024-02-28

**Authors:** Oleksandr Sverdlov, Yevgen Ryeznik, Volodymyr Anisimov, Olga M. Kuznetsova, Ruth Knight, Kerstine Carter, Sonja Drescher, Wenle Zhao

**Affiliations:** 1 Novartis Pharmaceuticals Corporation, East Hanover, NJ USA; 2 Department of Pharmacy, Uppsala University, Uppsala, Sweden; 3 Amgen Ltd., London, UK; 4 Merck & Co., Inc., Rahway, NJ USA; 5 Liverpool Clinical Trials Centre, University of Liverpool, Merseyside, Liverpool, UK; 6 Boehringer-Ingelheim Pharmaceuticals Inc, Ridgefield, CT USA; 7 Boehringer-Ingelheim Pharma GmbH & Co. KG, Biberach, Germany; 8 Medical University of South Carolina, Charleston, SC USA

**Keywords:** Multi-center clinical trial, Maximum tolerated imbalance, Allocation randomness, Poisson-gamma model, Recruitment time

## Abstract

**Background:**

The design of a multi-center randomized controlled trial (RCT) involves multiple considerations, such as the choice of the sample size, the number of centers and their geographic location, the strategy for recruitment of study participants, amongst others. There are plenty of methods to sequentially randomize patients in a multi-center RCT, with or without considering stratification factors. The goal of this paper is to perform a systematic assessment of such randomization methods for a multi-center 1:1 RCT assuming a competitive policy for the patient recruitment process.

**Methods:**

We considered a Poisson-gamma model for the patient recruitment process with a uniform distribution of center activation times. We investigated 16 randomization methods (4 unstratified, 4 region-stratified, 4 center-stratified, 3 dynamic balancing randomization (DBR), and a complete randomization design) to sequentially randomize $n=500$ patients. Statistical properties of the recruitment process and the randomization procedures were assessed using Monte Carlo simulations. The operating characteristics included time to complete recruitment, number of centers that recruited a given number of patients, several measures of treatment imbalance and estimation efficiency under a linear model for the response, the expected proportions of correct guesses under two different guessing strategies, and the expected proportion of deterministic assignments in the allocation sequence.

**Results:**

Maximum tolerated imbalance (MTI) randomization methods such as big stick design, Ehrenfest urn design, and block urn design result in a better balance–randomness tradeoff than the conventional permuted block design (PBD) with or without stratification. Unstratified randomization, region-stratified randomization, and center-stratified randomization provide control of imbalance at a chosen level (trial, region, or center) but may fail to achieve balance at the other two levels. By contrast, DBR does a very good job controlling imbalance at all 3 levels while maintaining the randomized nature of treatment allocation. Adding more centers into the study helps accelerate the recruitment process but at the expense of increasing the number of centers that recruit very few (or no) patients—which may increase center-level imbalances for center-stratified and DBR procedures. Increasing the block size or the MTI threshold(s) may help obtain designs with improved randomness–balance tradeoff.

**Conclusions:**

The choice of a randomization method is an important component of planning a multi-center RCT. Dynamic balancing randomization with carefully chosen MTI thresholds could be a very good strategy for trials with the competitive policy for patient recruitment.

**Supplementary Information:**

The online version contains supplementary material available at 10.1186/s12874-023-02131-z.

## Introduction

The randomized controlled trial (RCT) is the hallmark research methodology for evaluating which (if any) of two or more treatments is more effective [1]. In the simplest case, the design of a 1:1 RCT involves quantifying study objectives and determining the required sample size ($n$) to detect a statistically significant difference between the experimental and control groups for the assumed trial parameters. For the chosen $n$, randomization is applied to sequentially randomize eligible participants to either experimental or control group, and after the target number of participants are randomized, treated, and assessed for a defined period, their primary outcomes are analyzed to test the pre-specified clinical research hypothesis.

In practice, the design of RCTs may involve additional important considerations, such as utilization of more than one research site (center) for study conduct, stochastic nature of the study participant recruitment process, and stratification of the design by some important prognostic factors, including center [2]. A multi-center RCT model has several advantages. It enables a more inclusive experiment with broader coverage of patients across different geographies and it can expedite the recruitment of the target number of study participants. For some indications, such as rare diseases, using multiple centers may be the only feasible way to implement the study. At the same time, there are some important methodological and practical issues with the multi-center RCT model [3]. At the study planning stage, the number of centers is typically pre-determined based on the budgetary considerations and expected sites’ capacities for enrolling and treating patients. However, during the trial conduct some centers that are unable to recruit any patients may be terminated, and new centers may be added to fulfil the recruitment targets. In practice, study centers are activated over time, and they may have different capabilities to recruit and retain patients; therefore, the number of participants per center is, in general, a random variable. Stochastic models for patient recruitment and prediction of enrollment in multi-center RCTs are increasingly used in practice [4, 5].

Randomization—the assignment of treatments to study participants using a chance procedure—is an essential component of any RCT, and the choice of a randomization method for a given trial may not be simple [6, 7]. One important consideration is the choice between unstratified and stratified randomization. *Unstratified* randomization means that eligible participants are randomized to treatment arms according to a single randomization schedule that can be pre-generated before the trial starts. Different restricted randomization procedures can be used to produce a randomization sequence with equal or nearly equal numbers of treatment assignments [8]; however, in a multi-center RCT unstratified randomization does not guarantee that treatment numbers are balanced within study centers, and this may add to variability in the study results and may cause an additional level of uncertainly for drug supply planning [9].

*Stratified* randomization means that eligible participants are grouped according to baseline covariate values into mutually exclusive strata prior to randomization, and within each stratum a separate randomization sequence is used to allocate participants to treatments. Stratified randomization can be used in RCTs for both statistical and pragmatic reasons [10]. In multi-center RCTs, study site/center may be considered as a stratification factor, e.g., to optimize the use of drug supply and/or avoid confounding of the center effect with other known or unknown prognostic factors [2, 11]. While potentially useful in some circumstances, center-stratified randomization may lead to an undesirable imbalance in treatment group sizes when there are too many study sites/centers with very few patients per center. Another possibility is to use geographic region as a stratification factor [11, 12]. In this case, study centers within the same region belong to the same stratum, and treatment assignments of the participants enrolled by these centers are determined based on the corresponding stratum-specific randomization sequence. The 2015 EMA guideline on adjustment for baseline covariates has the following text in this regard [11]:

“…If a multicentre trial is not stratified by centre (e.g., when the number of patients within many centres is expected to be very small), it should be considered whether randomisation could be stratified by, for example, country or region. Such a choice might be driven by similarities in co-medication, palliative care or other factors that might make stratification advisable. The reasons and justification for the choice should be described in the protocol.”

Furthermore, stratification can be done by some prognostic baseline covariates (e.g., sex, age, disease severity, etc.) that are thought to be strongly related to the primary outcome, or their combination with administrative factors (center or geographic region). One should be always mindful of the total number of the resulting strata which can be overwhelming [13, 14]. The properties of imbalance caused by center-stratified permuted-block randomization and its impact on the power and sample size of the study were investigated in the papers [15–19].

Once the set of stratification factors has been decided upon, the next important consideration is the choice of a randomization method within strata. The most common approach is the stratified permuted block design (PBD), for which sequences of permuted blocks of a fixed or random length are generated independently within strata. Since the number of participants per site/per stratum is generally random, there is a potential of treatment imbalance due to unfilled last blocks in some strata, which can be aggregated at the trial level, and this may lead to a sizable imbalance at the trial level if several strata have imbalance in favor of the same treatment. Choosing a small block size ensures better balance but increases the predictability of the allocation sequence and the risk of selection bias in open-label trials. One can replace PBD within strata with some less restrictive (and, therefore, less predictable) randomization method, such as maximum tolerated imbalance (MTI) randomization [20–22].

Alternatively, instead of stratified randomization one can use one of the dynamic covariate-adaptive allocation methods that provide balance within centers and/or regions, such as dynamic balanced randomization (DBR) [23, 24], minimization [25, 26], modified Zelen’s approach [27, 28] or some other covariate-adaptive randomization method. However, identifying the most appropriate method for a given trial setting requires careful evaluation of different design options.

The goal of the current paper is to perform a systematic assessment of randomization methods for a multi-center 1:1 RCT assuming a stochastic recruitment model. We use Monte Carlo simulations for a head-to-head comparison of various randomization designs under different trial assumptions to provide recommendations on the choice of a randomization method that achieves “best” performance in terms of balance and randomness criteria. The investigation of statistical inference criteria (e.g., power and validity of significance tests) is beyond the scope of the current work. Note that balance and randomness criteria provide some indirect measures of statistical efficiency and susceptibility to selection bias of randomization procedures.

The rest of the paper is organized as follows. The “Methods” section provides some background on the stochastic recruitment (Poisson-gamma) model, different randomization methods for a multi-center 1:1 RCT and describes a simulation study setup. The “Results” section presents the results of Monte Carlo simulations comparing 16 different randomization designs in terms of balance and randomness criteria. The “Conclusions” section summarizes the key findings, provides some practical recommendations on the choice of fit for purpose randomization methods for the considered experimental settings, and outlines some future work.

## Methods

In this section, we first describe a model for patient recruitment and randomization in the context of multi-center RCTs. The model will allow us to link several relevant sources of uncertainty—the variation in recruitment rates across study centers, different numbers of subjects recruited per center due to the competitive recruitment policy, and the random number of treatment assignments based on the chosen randomization method. We will also describe different measures of balance and randomness to quantify the statistical performance of various randomization designs and a Monte Carlo simulation study setup.

### Stochastic recruitment model

Consider a multi-center study which is designed to recruit $n$ patients from $N$ centers. There are three different policies for patient recruitment [29]: 1) *competitive recruitment*, for which there is no restriction on the number of patients recruited per center; 2) *balanced recruitment*, which assumes waiting until the number of patients in each center reaches some fixed value ${n}_{0}=n/N$; and 3) *restricted recruitment*, which assumes that every center must enroll at least ${n}_{*}$ patients and cannot enroll more than ${n}^{*}$ patients, where ${n}_{*}$ and ${n}^{*}$ are given threshold values. Throughout the paper, we assume the competitive recruitment policy, which is most realistic in practice. The target time to complete the recruitment is set to $T>0$. The actual recruitment time, $T(n,N)$, is a random variable. In practice, the values $(n,N)$ may be chosen based on statistical and budgetary considerations as a solution to some formal optimization problem [29–31]. One may require that ${\text{Pr}}\left(T(n,N)<T\right)\ge p$ for some pre-specified value of $p$, e.g., $p=0.90$.

For the patient recruitment process, we assume a Poisson-gamma model [17, 29, 30]. The patients arrive at different centers according to independent Poisson processes with some rates. As different centers have different capacity and recruitment speed, the recruitment rates are modelled using a gamma distribution. Centers can also be activated at different times.

Let us describe the recruitment processes in clinical centers: consider a trial with $N$ centers (located in $G$ geographic regions) that are activated independently over time. For the $i$ th center ($i=1,\dots ,N$), the center activation time is ${u}_{i}\ge 0$, which can be either fixed or random. For this center, the recruitment follows a Poisson process with the recruitment rate ${\lambda }_{i}$ which is assumed to follow the gamma distribution, i.e., ${\lambda }_{i}\sim Gamma(\alpha ,\beta )$ with probability density function (p.d.f.) $p\left(x|\alpha ,\beta \right)=\frac{{\beta }^{\alpha }}{\Gamma \left(\alpha \right)}{x}^{\alpha -1}{e}^{-\beta x}$, $x>0$, where $\alpha ,\beta >0$ are the hyperparameters defined at the study planning stage. With this parametrization, $E{\lambda }_{i}=\frac{\alpha }{\beta }$ and ${var \lambda }_{i}=\frac{\alpha }{{\beta }^{2}}$.

Let ${n}_{i}(t)$ be the number of patients recruited at the $i$th center by time $t>0$. Consider first the “idealized” scenario when all $N$ centers are activated simultaneously at time zero, i.e., ${u}_{i}\equiv 0$ for $i=1,\dots ,N$. In this case, ${n}_{i}(t)$ is a mixed Poisson process (i.e., Poisson-gamma process) with random rate ${\lambda }_{i}$, and for any fixed $t$, the variable ${n}_{i}(t)$ has a negative binomial distribution ($NBin$) with parameters $(\alpha ,\frac{t}{\beta })$ such that $E\left[{n}_{i}(t)\right]=\frac{\alpha t}{\beta }$ and $var\left[{n}_{i}(t)\right]=\frac{\alpha t}{\beta }+\frac{\alpha {t}^{2}}{{\beta }^{2}}$. Furthermore, the global recruitment $n\left(t\right)={\sum }_{i=1}^{N}{n}_{i}(t)$ is also a mixed Poisson process with random rate $\Lambda ={\sum }_{i=1}^{N}{\lambda }_{i}$ and for any fixed $t$, $n\left(t\right)\sim NBin(\alpha N,\frac{t}{\beta })$. The recruitment time $T\left(n,N\right)$ has a Pearson type VI distribution, and its p.d.f., mean, and variance can be found in [30]. Finally, let ${\varvec{n}}=({n}_{1},\dots ,{n}_{N})$ denote the vector of the number of patients recruited by different centers at time $T\left(n,N\right)$, where $0\le {n}_{i}\le n$ and ${\sum }_{i=1}^{N}{n}_{i}=n$. Set $\Lambda ={\sum }_{i=1}^{N}{\lambda }_{i}$, ${p}_{i}=\frac{{\lambda }_{i}}{\Lambda }$, and ${\varvec{p}}=({p}_{1},\dots ,{p}_{N})$. Then the conditional distribution of ${\varvec{n}}|{\varvec{p}}$ is multinomial with parameters $(n,{\varvec{p}})$, the unconditional distribution of vector ${\varvec{n}}$ is Dirichlet-multinomial, and the marginal distributions of ${n}_{i}$’s are Beta-binomial:

$${\text{Pr}}\left({n}_{i}=j\right)=\left(\begin{array}{c}n\\ j\end{array}\right)E\left[{p}_{i}^{j}{\left(1-{p}_{i}\right)}^{n-j}\right], j=\mathrm{0,1},\dots ,n,$$

where $${p}_{i}\sim Beta(\alpha ,\alpha N-\alpha )$$, $$i=1,\dots ,N$$ [32].

In practice, it is unrealistic for all centers to be activated at once. It is more plausible to consider a stochastic process for center activation over time. Suppose that centers are activated with delay, i.e., ${u}_{i}$’s are random variables; for instance, ${u}_{i}\sim Uniform({a}^{\prime},{a}^{{\prime}{\prime}})$, where the constants $0\le {a}^{\prime}<{a}^{{\prime}{\prime}}$ are defined at the planning stage. Then ${n}_{i}(t)$ is a non-homogeneous Poisson process with cumulative rate on the interval $[0,t]$ equal to ${\lambda }_{i}\left(t\right)={\lambda }_{i}\left(t-{u}_{i}\right)\chi ({u}_{i}\le t)$, where $\chi (A)$ stands for the indicator of the event $A$. Also, $n\left(t\right)$ is a non-homogeneous Poisson process with cumulative rate $\Lambda \left(t\right)={\sum }_{i=1}^{N}{\lambda }_{i}\left(t-{u}_{i}\right)\chi ({u}_{i}\le t)$. In the case of a random center’s activation, uniform distribution was considered in [33], and a more general case of using beta and gamma distributions in [34]. However, characterizing the randomization process on the top of the recruitment process analytically is difficult, therefore we use Monte Carlo simulation.

### Randomization

#### Unstratified randomization

For a 1:1 RCT with $n$ sequentially enrolled patients, a *randomization sequence* is a random vector ${{\varvec{\Delta}}}_{n}=({\delta }_{1},\dots ,{\delta }_{n})$, where ${\delta }_{m}=1$ (or 0), if the $m$ th patient in the sequence is randomized to treatment E (or treatment C). The simplest procedure is complete (a.k.a. “simple”, “unrestricted”) randomization for which any participant is randomized to E or C with probability 0.5, i.e., the elements of ${{\varvec{\Delta}}}_{n}$ are independent Bernoulli(0.5) random variables [35]. A major limitation of complete randomization is that it can result, with non-negligible probability, in deviations from the 1:1 target allocation. In practice, some restrictions on randomization are applied. A *restricted* randomization procedure aims at balancing treatment assignments over time according to the target allocation ratio (e.g., 1:1), and it can be defined by specifying the conditional randomization probability of the ($m+1$)^st^ patient to treatment E given the past treatment assignments:

1 $${\phi }_{m+1}={\text{Pr}}\left({\delta }_{m+1}=1|{{\varvec{\Delta}}}_{m}\right), 1\le m\le n-1;and\; {\phi }_{1}=0.5.$$

Note that with unstratified restricted randomization, the patients in the sequence may come from different study sites and different geographic regions, but this information is irrelevant to their treatment assignment. Let ${n}_{E}\left(m\right)={\sum }_{l=1}^{m}{\delta }_{l}$ and ${n}_{C}\left(m\right)=m-{n}_{E}\left(m\right)$ denote the number of patients randomized to E and C, respectively after $m$ allocations, and let $D\left(m\right)={n}_{E}\left(m\right)-{n}_{C}\left(m\right)$ denote the treatment imbalance. For many unstratified restricted randomization procedures, the allocation rule (1) is expressed in the form

2 $${\phi }_{m+1}=F\left(D(m)\right),$$

where $F(\cdot )$ is some nonincreasing, symmetric around zero function of imbalance in treatment assignments. We shall consider four restricted randomization procedures with different form of $F(\cdot )$, all of which have the maximum tolerated imbalance (MTI) property, i.e., for some pre-specified small positive integer $b$, $\left|D(m)\right|\le b$ for any allocation step $m\ge 1$. The procedures are:

- *Permuted block design (PBD)* [36]: treatment assignments are made at random in blocks of $2b$ (exactly $b$ assignments to each treatment E and C in each block).
- *Big stick design (BSD)* [37]: every subject is randomized to E or C with probability 0.5 as long as treatment imbalance is less than $b>0$; if $\left|D(j)\right|=b$, the next allocation is made deterministically to the underrepresented treatment to restore near-balance within acceptable limits.
- *Ehrenfest urn design (EUD)* [38]: Consider two urns representing treatment groups E and C. There are $2b$ balls, initially equally distributed between the urns. For a given subject, a ball is drawn at random from the pool of $2b$ balls. The selection of a ball from urn $k$ corresponds to the assignment of treatment $k=E,C$. The chosen ball is then placed into the opposite urn. The described steps are repeated for the next subject.
- *Block urn design (BUD)* [39]: Consider two urns: active and inactive. Initially the active urn contains $2b$ balls ($b$ balls of each type E or C) and the inactive urn is empty. For a given subject, a ball is drawn at random from the active urn. If type $k$ ball is drawn ($k=E,C$), then treatment $k$ is assigned and the ball is placed in the inactive urn. The procedure is repeated until one type E ball and one type C ball appear in the inactive urn, in which case these two balls are placed into the active urn. The described steps are repeated for the next subject.

The formulas for the function $F(\cdot )$ of PBD, BSD, EUD, and BUD are as follows:

$$\mathrm{PBD}:\;\;0.5\left(1-\frac{D\left(m\right)}{2b+2b\cdot int\left(\frac m{2b}\right)-m}\right)$$

$$\mathrm{BSD}:\;\;0.5\left(1-sign\left(D\left(m\right)\right)\cdot int\left(\frac{\left|D\left(m\right)\right|}b\right)\right)$$

$$\mathrm{EUD}:\;\;0.5\left(1-\frac{D\left(m\right)}b\right)$$

$$\mathrm{BUD}:\;\;0.5\left(1-\frac{D\left(m\right)}{2b-\left|D(m)\right|}\right)$$

In the above, $int(x)$ stands for the function that returns an integer less than or equal to $x$, and $sign(x)$ stands for the function that returns value -1, 0, 1 if $x$ is negative, zero, or positive, respectively.

#### Stratified randomization

Stratified randomization utilizes independent restricted randomization procedures within $M$ mutually exclusive strata defined by all possible combinations of the given factor levels. Let ${z}_{l}$ denote the stratum ID for the $l$ th patient (${z}_{l}=s$, if the $l$th patient belongs to stratum $s$, where $s=1,\dots ,S$). In practice, ${\left\{{z}_{l}\right\}}_{l\ge 1}$ is possibly a random sequence. The first patient in each stratum is randomized to treatment E or C with probability 0.5. Subsequent treatment assignments within a stratum are made conditional on the past treatment assignments in that stratum. Let ${{\varvec{z}}}_{m}=({z}_{1},\dots ,{z}_{m})$ be the information on the strata of first $m$ patients in the study and suppose ${z}_{m+1}=s$ is the stratum ID of the next, ($m+1$)^st^ patient. Let ${n}^{(s)}(m)={\sum }_{l=1}^{m}\chi ({z}_{l}=s)$ be the total number of patients in the $s$th stratum among the $m$ patients in the study, and ${{\varvec{\Delta}}}_{m}^{(s)}$ be the corresponding vector of treatment assignments of ${n}^{(s)}(m)$ patients in the $s$th stratum. Note that since ${n}^{(s)}(m)$ is random, the vector ${{\varvec{\Delta}}}_{m}^{(s)}$ has a random length. The conditional randomization probability for the ($m+1$)^st^ patient belonging to stratum $s$ is expressed as ${\phi }_{m+1}={\text{Pr}}\left({\delta }_{m+1}=1|{{\varvec{\Delta}}}_{m}^{(s)}\right), m\ge 1$.

Let ${n}_{k}^{(s)}(m)$ be the number of patients in the $s$th stratum randomized to treatment $k=E,C$, and ${D}^{(s)}\left(m\right)={n}_{E}^{(s)}(m)-{n}_{C}^{(s)}(m)$ be the imbalance in the $s$th stratum after $m$ allocation steps. Then, the allocation rule for the ($m+1$)^st^ patient can be formulated as

3 $$\phi_{m+1}=\sum\nolimits_{s=1}^SF(D^{(s)}\left(m\right))\cdot\chi(z_{m+1}=s)$$

where $F(\cdot )$ is a pre-specified allocation function of some restricted randomization procedure, such as PBD, BSD, EUD, or BUD (cf. *Unstratified randomization*). Note that since the strata are mutually exclusive, only one term in the sum at the right-hand side of Eq. (3) will be non-zero, i.e., the term $F({D}^{(s)}\left(m\right))$ that corresponds to the stratum $s$ of the ($m+1$)^st^ patient.

Next, we introduce some notations for the two special cases—when the randomization is stratified by center, and when it is stratified by region—to distinguish different types of treatment counts and imbalances within the strata. Suppose the stratification variable is the study center. For the $i$th center ($i=1,\dots ,N$), define ${n}_{i}(m)=$ number of patients recruited by the $i$th center, ${n}_{i,k}(m)=$ number of patients in the $i$th center randomized to treatment $k=E,C$, and ${D}_{i}\left(m\right)={n}_{i,E}\left(m\right)-{n}_{i,C}\left(m\right)=$ imbalance in the $i$th center after $m$ allocation steps. For the four center-stratified randomization procedures considered in this paper, the allocation rule (3) becomes ${\phi }_{m+1}={\sum }_{i=1}^{N}F\left({D}_{i}\left(m\right)\right)\cdot \chi ({z}_{m+1}=i)$, where $F(\cdot )$ is the pre-specified allocation function (cf. *Unstratified randomization*). With center-stratified randomization, it is expected that the final imbalance within center, ${D}_{i}\left(n\right)$, is close to 0 for every $i=1,\dots ,N$.

Suppose the stratification variable is the geographic region. For the $g$th region ($g=1,\dots ,G$), define ${\widetilde{n}}_{g}\left(m\right)={\sum }_{i\in {I}_{g}}{n}_{i}\left(m\right)=$ number of patients recruited by the centers located in region $g$, ${\widetilde{n}}_{g,k}\left(m\right)={\sum }_{i\in {I}_{g}}{n}_{i,k}\left(m\right)=$ number of patients recruited in region $g$ that are randomized to treatment $k=E,C$, and ${\widetilde{D}}_{g}\left(m\right)={\widetilde{n}}_{g,E}\left(m\right)-{\widetilde{n}}_{g,C}\left(m\right)=$ treatment imbalance in region $g$ after $m$ allocation steps. For the four region-stratified randomization procedures considered in this paper, the allocation rule (3) for the ($m+1$)^st^ patient (assuming the patient is recruited in region $g$) is cast as ${\phi }_{m+1}={\sum }_{g=1}^{G}F({\widetilde{D}}_{g}\left(m\right))\cdot \chi ({\widetilde{z}}_{m+1}=g)$, where $F(\cdot )$ is the pre-specified allocation function (cf. *Unstratified randomization*). With region-stratified randomization, it is expected that the final within-region imbalance, ${\widetilde{D}}_{g}\left(n\right)$, is close to 0 for every $g=1,\dots ,G$.

#### Dynamic balancing randomization

Dynamic balancing randomization (DBR) is a covariate-adaptive randomization method proposed by Signorini and co-authors [23]; see also references [24, 40]. It attempts to sequentially balance treatment assignments according to a pre-specified hierarchy of classification factors. With DBR, it is not possible to pre-generate the randomization sequence, and so it is created dynamically, depending on the imbalances within the observed levels of the factors of the new patient. To contextualize DBR for our example, let ${b}_{1}, {b}_{2},{b}_{3}$ be some pre-specified positive integers that define the “acceptable” limits for treatment imbalance at the center, region, and trial level. Consider a point in the trial when $m$ patients have been randomized, and based on the data (${{\varvec{\Delta}}}_{m}$, ${{\varvec{z}}}_{m}$, ${\widetilde{{\varvec{z}}}}_{m}$), we have the values of imbalances at the center level: ${D}_{i}\left(m\right) (i=1,\dots ,N)$, region level: ${\widetilde{D}}_{g}\left(m\right)$ $(g=1,\dots ,G)$, and trial level: $D(m)$. Suppose the $(m+1)^\mathrm{st}$ patient is recruited at center $i$ located within geographic region $g$ (i.e., ${z}_{m+1}=i$ and ${\widetilde{z}}_{m+1}=g$). Then the DBR algorithm to determine the treatment assignment for the $(m+1)^\mathrm{st}$ patient involves the following steps:

- Step 1 (balance at the center level): If $\left|{D}_{i}\left(m\right)\right|={b}_{1}$, then choose the treatment assignment deterministically, to reduce imbalance within this center; otherwise go to Step 2.
- Step 2 (balance at the region level): If $\left|{\widetilde{D}}_{g}\left(m\right)\right|\ge {b}_{2}$, then choose the treatment assignment deterministically, to reduce imbalance within this region; otherwise go to Step 3.
- Step 3 (balance at the trial level): If $\left|D(m)\right|\ge {b}_{3}$, then choose the treatment assignment deterministically, to reduce imbalance at the trial level; otherwise go to Step 4.
- Step 4: Allocate a treatment at random: ${\phi }_{m+1}=0.5$.

The DBR can be thought of as a covariate-adaptive extension of the big stick design [37]. One important practical question is the choice of imbalance thresholds ${b}_{1}, {b}_{2},{b}_{3}$ that would provide a sensible tradeoff between treatment balance and allocation randomness.

### Measures of balance and randomness

Balance and randomness are two competing requirements for any RCT. Restricted randomization is applied to balance treatment assignments (overall in the study or/and within baseline covariate strata) while maintaining the randomized nature of the experiment. Balanced allocation is desirable from the standpoints of statistical efficiency and drug supply management. There are different ways of quantifying imbalance. Here we focus on the end-of-enrollment measures, calculated after $n$ patients have been randomized in the study.

To understand why treatment balance is important, it is instructive to consider a statistical model and the concept of loss [41, 42]. Suppose the responses of $n$ patients in the trial (conditional on the treatment assignments and the selected covariates) satisfy a normal linear model.

4 $${\varvec{Y}}={\varvec{Z}}^{\prime}{\varvec{\beta}}+\alpha {\varvec{t}}+{\varvec{\varepsilon}},$$

where ${\varvec{Y}}$ is $n\times 1$ vector of responses and ${\varvec{\varepsilon}}\sim {\varvec{N}}(0,{\sigma }^{2}{\varvec{I}})$ is a vector of error terms. The design matrix for model (4) is of the form ${\varvec{X}}=[{\varvec{Z}\;}\boldsymbol{ }{\varvec{t}}]$, where ${\varvec{Z}}$ is an $n\times p$ matrix of covariates (including the intercept), and ${\varvec{t}}$ is an $n\times 1$ vector of treatment assignment indicators (${t}_{m}=$ 1 or -1 for treatment E or C, $m=1,\dots ,n$). Here, we use ${t}_{m}$ instead of ${\delta }_{m}$ for mathematical convenience; a simple transformation can be applied: ${t}_{m}=2{\delta }_{m}-1$.

The vector of model parameters is ${\varvec{\theta}}=({\varvec{\beta}},\alpha )$, and the primary interest is the estimation of the treatment effect $\alpha$. The least squares estimator of ${\varvec{\theta}}$ is $\widehat{{\varvec{\theta}}}={\left({\varvec{X}}^{\prime}{\varvec{X}}\right)}^{-1}{\varvec{X}}^{\prime}{\varvec{Y}}$ with variance–covariance matrix ${\sigma }^{2}{\left({\varvec{X}}^{\prime}{\varvec{X}}\right)}^{-1}$**.** The variance of $\widehat{\alpha }$ is the lower diagonal element of this matrix, expressed as

5 $$var\left(\widehat{\alpha }\right)=\frac{{\sigma }^{2}}{n-{{\varvec{t}}}^{\prime}{\varvec{Z}}{\left({{\varvec{Z}}}^{\prime}{\varvec{Z}}\right)}^{-1}{{\varvec{Z}}}^{\prime}{\varvec{t}}}$$

The second term in the denominator is referred to as *loss* [41]:

6 $$L={\varvec{t}}^{\prime}{\varvec{Z}}{\left({\varvec{Z}}^{\prime}{\varvec{Z}}\right)}^{-1}{\varvec{Z}}^{\prime}{\varvec{t}}$$

The loss can be thought of as the number of patients in the study from whom information is lost due to randomization-induced treatment imbalance compared to the “idealized” balanced design (note that $var\left(\widehat{\alpha }\right)$ in (5) is minimized when (6) is equal to zero, which means that ${\varvec{t}}$ is orthogonal to the columns of ${\varvec{Z}}$ that are assumed to be fixed, i.e., non-stochastic**)**. Depending on the structure of ${\varvec{Z}}$, one can have different forms of (6). For instance, if ${\varvec{Z}}=\boldsymbol{1}$, a single column of 1’s, we have

7 $$L_1=\frac{\left(n_E\left(n\right)-n_C\left(n\right)\right)^2}n=\frac{\left\{D\left(n\right)\right\}^2}n.$$

Note that ${L}_{1}$ in (7) is a random variable whose distribution is determined by the randomization procedure used in the study. Since for most 1:1 randomization procedures we have $E\left[D\left(n\right)\right]=0$, the expected value of (7) is $\frac{E{\left\{D\left(n\right)\right\}}^{2}}{n}=\frac{var\left[D\left(n\right)\right]\ }{n}$. Therefore, the efficiency in estimating the treatment effect is directly related to the variability of the randomization procedure—the most efficient procedure is one for which $var[D(n)]=0$ i.e., a randomization procedure that always results in final equal allocation per arm. Note that an MTI procedure ensures that $\left|D(n)\right|\le b$, and so ${L}_{1}\le \frac{{b}^{2}}{n}$, which is negligible for large $n$.

Suppose that geographic region is an important covariate that affects the response. Then ${\varvec{Z}}={{\varvec{Z}}}_{n\times G}=[{\boldsymbol1}\; {\widetilde{{\varvec{z}}}}_{1}\dots {\widetilde{{\varvec{z}}}}_{G-1}]$, where **1** is an intercept ($n\times 1$ vector of ones), ${\widetilde{{\varvec{z}}}}_{g}$ ($g=1,\dots ,G-1$) is an $n\times 1$ vector that has ${\widetilde{n}}_{g}$ entries equal to 1 (for those subjects who were recruited in region $g$) and the remaining $n-{\widetilde{n}}_{g}$ entries are equal to 0. In this case, the loss (6) is expressed as

8 $$L_2=\sum\nolimits_{g=1}^G\frac{\left\{{\widetilde D}_g\left(n\right)\right\}^2}{{\widetilde n}_g}.$$

If study center is an important covariate that affects the response, then we have ${\varvec{Z}}={{\varvec{Z}}}_{n\times N}=[{\boldsymbol1}\; {{\varvec{z}}}_{1}\dots {{\varvec{z}}}_{N-1}]$, where **1** is an intercept ($n\times 1$ vector of ones), ${{\varvec{z}}}_{i}$ ($i=1,\dots ,N-1$) is an $n\times 1$ vector that has ${n}_{i}$ entries equal to 1 (for those ${n}_{i}$ patients that have been recruited by center $i$) and the remaining $n-{n}_{i}$ entries equal to 0. In this case, the loss (6) has the form

9 $$L_3=\sum\nolimits_{i=1}^N\frac{\left\{D_i\left(n\right)\right\}^2}{n_i}$$

The proof of formulas (8) and (9) can be found in Supplemental Appendix 3.

We can also consider estimation efficiency of a particular randomization design with $var\left(\widehat{\alpha }\right)=\frac{{\sigma }^{2}}{n-L}$ relative to the “idealized” balanced design for which $var\left(\widehat{\alpha }\right)=\frac{{\sigma }^{2}}{n}$. We have

10 $$RE=\frac{\sigma^2/n}{\sigma^2/(n-L)}=1-\frac Ln.$$

where $L$ takes one of the forms (7), (8), or (9). $RE$ is a random variable taking values in the range 0–1 whose probability distribution can be evaluated using Monte Carlo simulations.

Another useful and easy-to-interpret measure is the standard deviation of absolute overall imbalance:

11 $$SD\left|D(n)\right|=\sqrt{var\left|D(n)\right|}$$

Some researchers (e.g., [43]) suggested using probabilistic measures to quantify the risk of imbalance. We consider probability distributions of imbalance at the trial, center, and region level; i.e., ${\text{Pr}}\left(\left|Imbalance\right|\ge d\right)$ for $d=\mathrm{0,1},2\dots$, where $\left|Imbalance\right|$ is equal to: $\left|D(n)\right|$ for the trial level; $\underset{i=1,\dots ,N}{{\text{max}}}\left|{D}_{i}\left(n\right)\right|$ for the center level; or $\underset{g=1,\dots ,G}{{\text{max}}}\left|{\widetilde{D}}_{g}\left(n\right)\right|$ for the region level.

Finally, to quantify how frequently a randomization design results in “extreme” allocation sequences at the center level, we propose the measure ${P}_{skewed}$, which is the expected proportion of centers (among all centers that recruited at least 2 patients) that resulted in the allocation ratio more skewed that 1:2 or 2:1. In other words, if for the $i$th center that recruited ${n}_{i}\ge 2$ patients, the absolute difference in treatment allocation proportions is > $1/3$, then the treatment allocation sequence for that center would be classified as “skewed”. More formally, we are interested in

12 $$P_{skewed}=E\left[\frac{\sum_{i=1}^N\chi\left(\frac{\left|D_i\left(n\right)\right|}{n_i}>\frac13\right)\cdot\chi\left(n_i\geq2\right)}{\sum_{i=1}^N\chi\left(n_i\geq2\right)}\right]$$

For quantifying lack of randomness in the study (which is directly linked to the selection bias), we consider the expected proportion of correct guesses under two different guessing strategies—the convergence strategy and the deterministic strategy [44], and the expected proportion of deterministic assignments in the sequence.

With the convergence guessing strategy, it is assumed that an investigator at the site/center level knows the number of treatment assignments in that center at any point in the trial, and applies the following intelligent guessing rule: Guess next treatment assignment in their center as E (or C), if the current number of allocations to E is less than (or greater than) the number of allocations to C (or E); otherwise make a random guess (with probability 0.5). With this approach, the expected proportion of correct guesses, $PC{G}_{c}$, is

13 $$PCG_c=\frac1n\sum\nolimits_{m=1}^nE\left(G_m\right)$$

where ${G}_{1}=0.5$ and for $m\ge 1$, ${G}_{m+1}$ is a random variable defined as follows:

14 $${G}_{m+1}=\left\{\begin{array}{ll}1,& if\; \left({D}_{i}\left(m\right)<0 \;and\; {\delta }_{m+1}=1\right) \;or\; \left({D}_{i}\left(m\right)>0\; and \;{\delta }_{m+1}=0\right);\\ 0.5,& if \;{D}_{i}\left(m\right)=0; \\ 0,& if\; \left({D}_{i}\left(m\right)<0 \;and \;{\delta }_{m+1}=0\right) \;or\; \left({D}_{i}\left(m\right)>0 \;and\; {\delta }_{m+1}=1\right).\end{array}\right.$$

Note that for the most random procedure, complete randomization design, $PC{G}_{c}=0.50$ regardless of the guessing strategy, and for any other (restricted) randomization procedure $PC{G}_{c}>0.50$. The selection bias is proportional to $PC{G}_{c}-0.5$.

With the deterministic guessing strategy, it is assumed that an investigator at the site/center level makes the (correct) guess only when the next treatment assignment in their center is known with certainty or guesses at random otherwise. With this approach, the expected proportion of correct guesses, $PC{G}_{d}$, is

15 $$PCG_d=\frac1n\sum\nolimits_{m=1}^nE\left({\widetilde G}_m\right)$$

where ${\widetilde{G}}_{m}$ is a random variable defined as follows:

16 $${\widetilde{G}}_{m}=\left\{\begin{array}{cc}1,& if {\phi }_{m}=0 \;or\; 1;\\ 0.5,& otherwise.\end{array}\right.$$

In (16), ${\phi }_{m}$ is the conditional randomization probability of treatment E assignment for the $m$th patient in the sequence, specific to the center at which this patient was recruited. For center-stratified MTI procedures—C-BSD, C-BUD, and C-EUD—the event $\left\{{\phi }_{m}=0 \;or\; 1\right\}$ occurs if and only if the current treatment imbalance has reached the MTI value, i.e., $\left|{D}_{i}(m-1)\right|=b$. For center-stratified PBD, deterministic allocations can occur more frequently than with MTI procedures, since more than a single assignment at the end of the block can be deterministic. Note that in our considered setting, the deterministic guessing strategy is meaningful only for center-stratified randomization and dynamic balancing randomization. For the unstratified and region-stratified randomization approaches (where the instants when deterministic allocations are made are unknown to an investigator at any given center), the deterministic guessing strategy is meaningless and for these procedures we can set $PC{G}_{d}\equiv 0.5$.

Finally, we consider the expected proportion of deterministic assignments in the sequence:

17 $$PD=\frac{1}{n}{\sum }_{m=1}^{n}{\text{Pr}}\left({\phi }_{m}=0 \;or\; 1\right)$$

Note that the terms in the sum in the right-hand side of (17) are considered without regard to the study center. The low value of $PD$ is desirable from the standpoint of statistical inference, because more random procedures have wider reference sets of randomization sequences and can result in potentially more powerful randomization tests [45].

### Simulation study setup

The goal of the Monte Carlo simulation is to compare several randomization strategies with respect to balance and randomness for a multi-center 1:1 RCT with a stochastic recruitment model. For the “base case” (Scenario 1), the following parameters will be used for the Poisson-gamma model:

- Target recruitment period $T=12$ months
- ${a}^{\prime}=0$*,* ${a}^{{\prime}{\prime}}=4$ (all centers are activated during the first 4-month period)
- $$n=500$$ patients
- $$N=80$$ centers
- $G=5$ regions (such that there are 16 centers per region)
- $$\alpha =1.2\times 100$$ and $$\beta =58\times 100$$

The latter choice of $\alpha$ and $\beta$ corresponds to a low variability of center recruitment rates and the goal of recruiting $n=500$ patients in the 1-year timeframe with the stated above recruitment parameters. To be more precise, assuming the total enrollment period $EP\approx 365$ days (12 months), and the center activation period $AP\approx 122$ days (4 months), the mean recruitment rate is $m=\frac{n}{\left(EP-\frac{AP}{2}\right)\cdot N}=\frac{500}{\left(365-61\right)\cdot 80}\approx 0.0206$ patients per center per day. Then by setting $\alpha =1.2\times 100$ and $\beta =\frac{\alpha }{m}\times 100$, we have the mean and variance of the recruitment rate for the $i$th center ($i=1,\dots ,80$) as $E\left({\lambda }_{i}\right)=\frac{\alpha }{\beta }\approx 0.0206$ and $var\left({\lambda }_{i}\right)=\frac{\alpha }{{\beta }^{2}}\approx 3.62\times {10}^{-6}$. This case reflects a very low variability in recruitment rates and is nearly equivalent to a Poisson model where in all centers the recruitment rates are constants. Though this is an idealistic situation, we consider is as a baseline scenario to compare with a more realistic Scenario 2 (see below) where the rates are random and have some reasonable variation.

We consider four approaches to randomization: i) unstratified; ii) center-stratified; iii) region-stratified; and iv) dynamic balancing randomization (DBR). For approaches i)–iii), four different methods to generate a randomization sequence are considered: permuted block design (PBD), big stick design (BSD), Ehrenfest urn design (EUD), and block urn design (BUD). These designs ensure that the absolute value of imbalance at the chosen level (trial, region, or center) is capped by a pre-specified positive integer MTI parameter $b$. For approach iv), there are 3 MTI parameters: ${b}_{1}$ (MTI parameter for the center level), ${b}_{2}$ (pursued but not always feasible MTI for the region level), and ${b}_{3}$ (pursued but not always feasible MTI for the trial level). Overall, 16 randomization methods will be compared:

I. Unstratified PBD $$(b=2)$$
II. Unstratified BUD $(b=2)$
III. Unstratified EUD $(b=2)$
IV. Unstratified BSD $(b=2)$
V. Region-stratified PBD $(b=2)$
VI. Region-stratified BUD $(b=2)$
VII. Region-stratified EUD $(b=2)$
VIII. Region-stratified BSD $(b=2)$
IX. Center-stratified PBD $(b=2)$
X. Center-stratified BUD $(b=2)$
XI. Center-stratified EUD $(b=2)$
XII. Center-stratified BSD $(b=2)$
XIII. DBR with ${b}_{1}=2$, ${b}_{2}=2$, ${b}_{3}=2$
XIV. DBR with ${b}_{1}=2$, ${b}_{2}=4$, ${b}_{3}=4$
XV. DBR with ${b}_{1}=2$, ${b}_{2}=4$, ${b}_{3}=8$
XVI. Complete randomization design (CRD)

The algorithm for simulation of patient recruitment and randomization is summarized in Table 1. In essence, for each simulation run, we first generate a recruitment pattern of $n$ patients in $N$ centers based on the assumed Poisson-gamma model. The resulting data structure, ${\mathcal{F}}_{n}$ will contain the following variables:

- Patient ID ($$m=1,\dots ,n$$)
- Patient enrollment times: ${t}_{m}\in [0,T]$, $m=1,\dots ,n$ (${t}_{1}\le {t}_{2}\le \dots \le {t}_{n}$)
- Patient enrollment centers: ${{\varvec{z}}}_{n}=({z}_{1},\dots ,{z}_{n})$
- Patient enrollment regions: ${\widetilde{{\varvec{z}}}}_{n}=({\widetilde{z}}_{1},\dots ,{\widetilde{z}}_{n})$ (the region is determined by the center)

**Table 1:** The algorithm for simulation of patient recruitment and randomization

Step 1	Specify the parameters of the trial (see subsection “Simulation Study setup”): $T, {a}^{{\prime}}, {a}^{{\prime}{\prime}},b, n, N, G, \alpha ,\beta$
Step 2	Generate the recruitment rates for the centers: *λ*_*i*_ ∼ *Gamma (α, β),* *i* = 1, …, *N*
Step 3	For the $\ell$th simulation run ($\ell=1,\dots ,\mathrm{10,000}$): a. For the $i$th center ($i=1,\dots ,N$): • Generate center activation time:${u}_{i}\sim Uniform({a}^{\prime},{a}^{{\prime}{\prime}})$ • Generate$n$patient arrival times according to the Poisson process with rate${\lambda }_{i}$ • Record the patient arrival times as$\left({u}_{i}+{t}_{i1}\right)\le \left({u}_{i}+{t}_{i2}\right)\le \dots \le ({u}_{i}+{t}_{in})$ b. Create a data structure of the pooled sample of $nN$ virtual patients with the following variables: • Patient ID ($l=1,\dots ,nN$) • Patient enrollment time (${\tau }_{l}={u}_{i}+{t}_{im}$,$i=1,\dots ,N$,$m=1,\dots ,n$) • Enrollment center • Geographic region of the center c. Sort the data structure from Step 3b by ${\tau }_{l}$, and retain the first $n$ patients $\Rightarrow$ call the resulting dataset ${\mathcal{F}}_{n}$ $\Rightarrow$it will constitute the sample of $n$ patients to be randomized in the $\ell$th simulation run d. Based on ${\mathcal{F}}_{n}$ from Step 3c, for each considered randomization design, generate: • Randomization sequence${{\varvec{\Delta}}}_{n}=({\delta }_{1},\dots ,{\delta }_{n})$, where ${\delta }_{m}=1$ or 0, if the $m$th patient in the sequence is randomized to treatment E or C • Vector${{\varvec{\Phi}}}_{n}=({\phi }_{1},\dots ,{\phi }_{n})$, where${\phi }_{m}$ is the conditional randomization probability of randomizing the $m$th patient in the sequence to treatment E
Step 4	Based on$\mathrm{10,000}$simulation runs, derive the operating characteristics, as described in subsection “Measures of balance and randomness”)

The simulated pattern ${\mathcal{F}}_{n}$ will be the same for different randomization methods, to ensure a consistent comparison. For a given randomization method, a randomization sequence ${{\varvec{\Delta}}}_{n}=({\delta }_{1},\dots ,{\delta }_{n})$ will be generated, accounting for the information from ${\mathcal{F}}_{n}$, as appropriate. Based on 10,000 simulations, we will obtain the measures of operational efficiency, measures of balance/statistical efficiency, and measures of randomness.

For the operational efficiency, we obtain:

- The distribution of time to complete recruitment (the time at which the $n$th patient has been enrolled into the study)
- The average number of centers that recruited exactly $j$ patients ($j=\mathrm{0,1},\dots ,n$).

For quantifying balance/statistical efficiency of each randomization method, we obtain:

- The distribution of loss at the trial level (${L}_{1}$), region level (${L}_{2}$), and center level (${L}_{3}$).
- The distribution of estimation efficiency of a randomization design relative to the idealized balanced design assuming a linear model with no covariates other than treatment (${RE}_{1}=1-{L}_{1}/n$), a linear model with additive effects of treatment and region (${RE}_{2}=1-{L}_{2}/n$), and a linear model with additive effects of treatment and center (${RE}_{3}=1-{L}_{3}/n$).
- The standard deviation of absolute imbalance, $SD\left|D(n)\right|$.
- The expected proportion of centers (among all centers that recruited at least 2 patients) that resulted in the allocation ratio more skewed that 1:2 or 2:1, ${P}_{skewed}$.
- The probability distributions of imbalance at the trial, center, and region level: ${\text{Pr}}\left(\left|Imbalance\right|\ge d\right)$ for $d=\mathrm{0,1},2\dots$, where $\left|Imbalance\right|$ is equal to: $\left|D(n)\right|$ for the trial level; $\underset{i=1,\dots ,N}{{\text{max}}}\left|{D}_{i}\left(n\right)\right|$ for the center level; or $\underset{g=1,\dots ,G}{{\text{max}}}\left|{\widetilde{D}}_{g}\left(n\right)\right|$ for the region level.

For quantifying the degree of randomness/predictability of each randomization method, we obtain:

- Average proportion of correct guesses assuming the convergence guessing strategy, $PC{G}_{c}$.
- Average proportion of correct guesses assuming the deterministic guessing strategy, $PC{G}_{d}$.
- Average proportion of deterministic assignments in the sequence, $PD$.

In addition to the described “base case” Scenario 1, we will investigate three more scenarios:

***Scenario 2:*** Increased variability in center recruitment rates: $\alpha =1.2$ and $\beta =58$ (which corresponds to $E\left({\lambda }_{i}\right)=\frac{\alpha }{\beta }\approx 0.0206$ and $var\left({\lambda }_{i}\right)=\frac{\alpha }{{\beta }^{2}}\approx 3.62\times {10}^{-4}$), and all other parameters as in Scenario 1. For this scenario, the recruitment rates have some realistic variation which is confirmed by the analysis of many real trials [30, 33].

***Scenario 3:*** Increased number of centers: $N=160$, and all other parameters as in Scenario 2.

***Scenario 4:*** Increased MTI threshold, and all other parameters as in Scenario 2. For all MTI randomization procedures, we set $b=4$, and for the three DBR procedures we set $\left({b}_{1},{b}_{2},{b}_{3}\right)=\left(4, 4, 4\right)$; $(4, 8, 8)$; $(4, 8, 16)$.

## Results

### Scenario 1

**Fig. 1:**
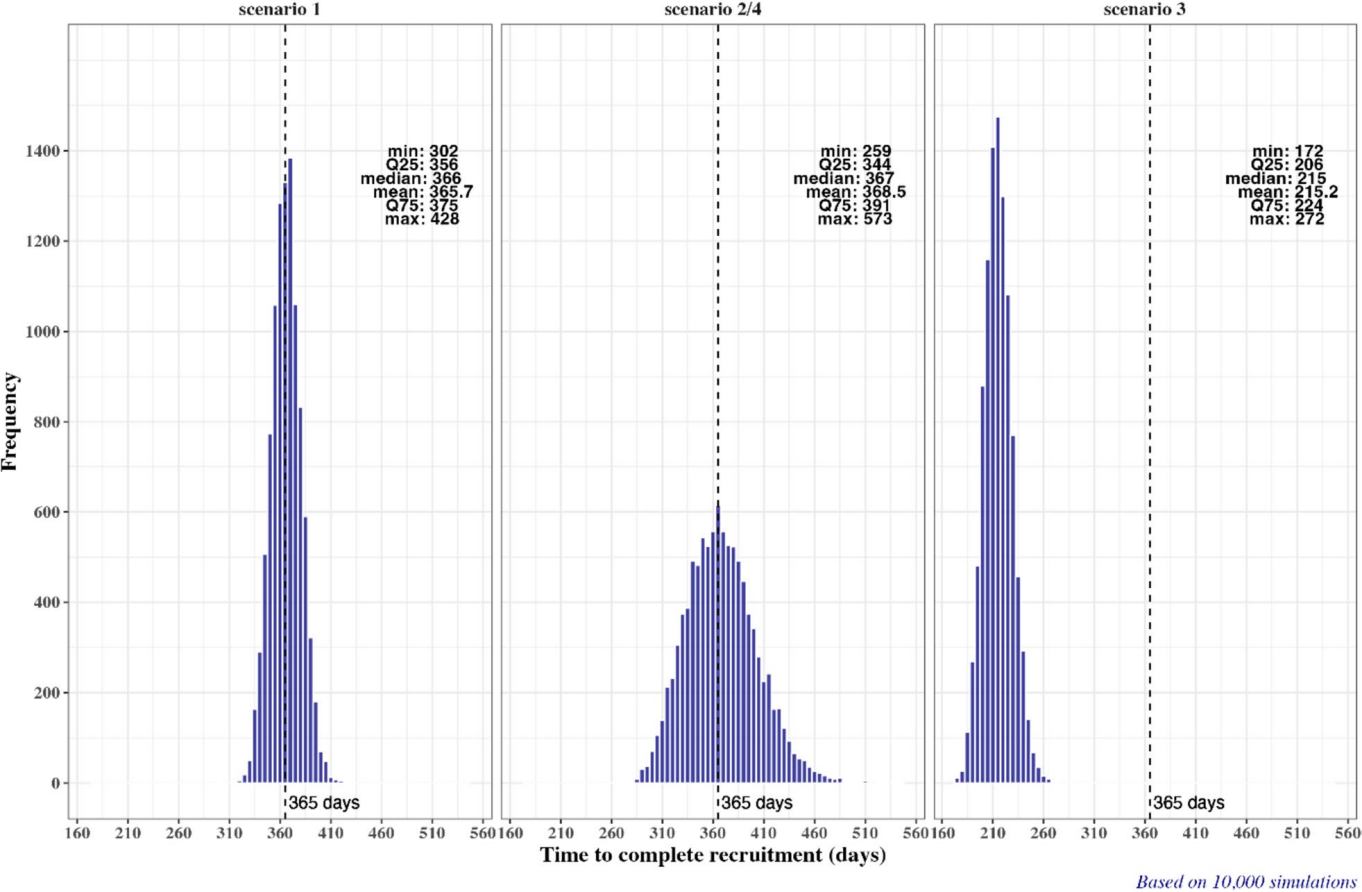
Simulated distribution of time to complete recruitment under Scenario 1 (left plot), under Scenarios 2 and 4 (middle plot), and under Scenario 3 (right plot)

Figure 1 (left plot) shows the simulated distribution of the time to complete recruitment by enrolling the 500th patient into the study. The distribution is symmetric and bell-shaped, centered around the target recruitment time of 365 days, with the interquartile range (IQR) from 356 to 375 days, and the range 302–428 days.

**Fig. 2:**
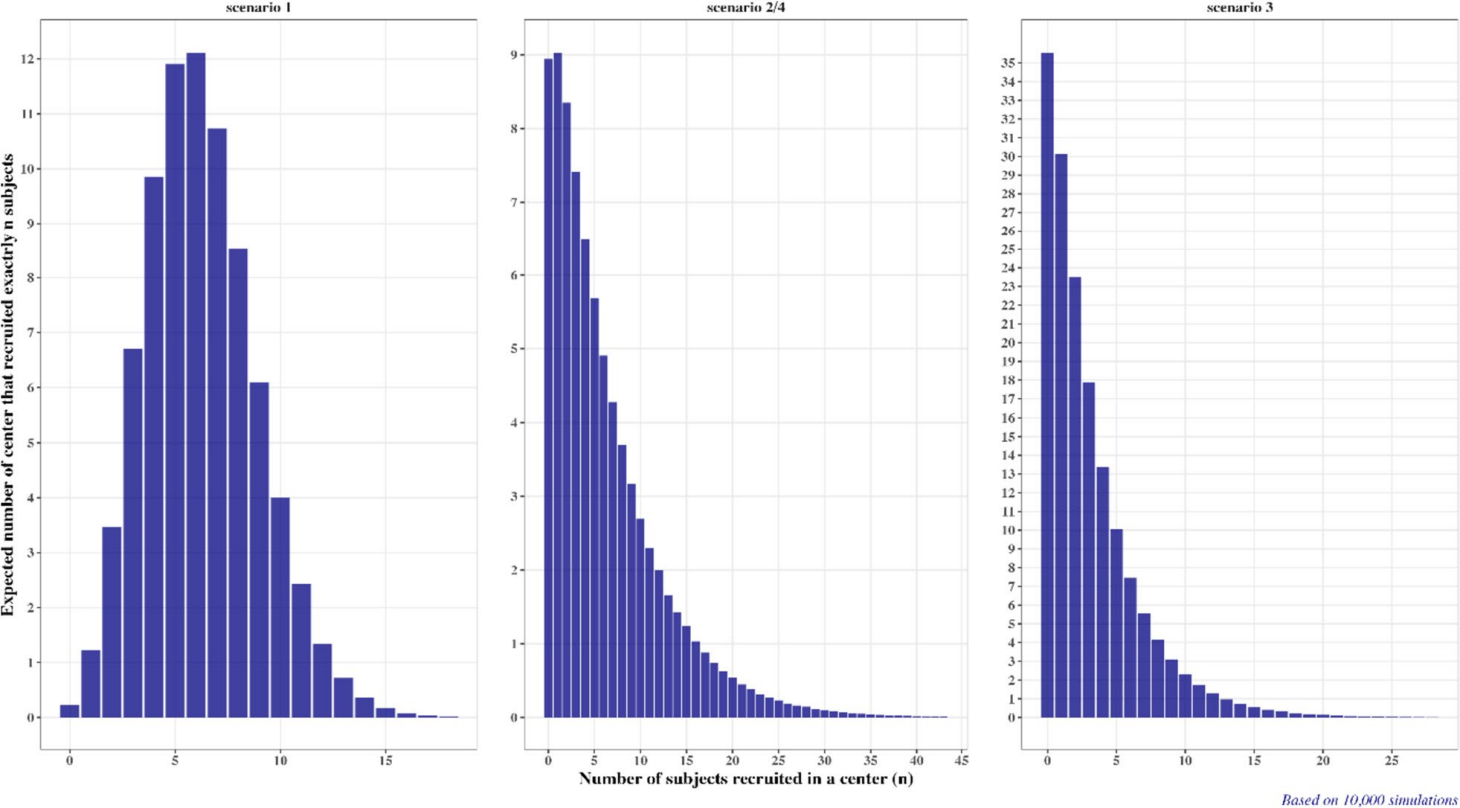
Simulated average number of centers that recruited exactly $j=\mathrm{0,1},\dots$ patients under Scenario 1 (left plot), Scenarios 2 and 4 (middle plot), and Scenario 3 (right plot)

Figure 2 (left plot) shows the simulated average number of centers that have recruited exactly $j=\mathrm{0,1},\dots$ patients. One can see that, on average, ~ 12 centers would recruit exactly 5 (or 6) patients, ~ 10 centers would recruit exactly 4 patients, etc. Also, less than one center, on average, would recruit exactly 13, 14 (or more) patients. This means that treatment imbalances at the center level are likely to occur—even if center-stratified randomization is used, some sites will recruit an odd number of patients, in which case the imbalance will be non-zero.

**Fig. 3:**
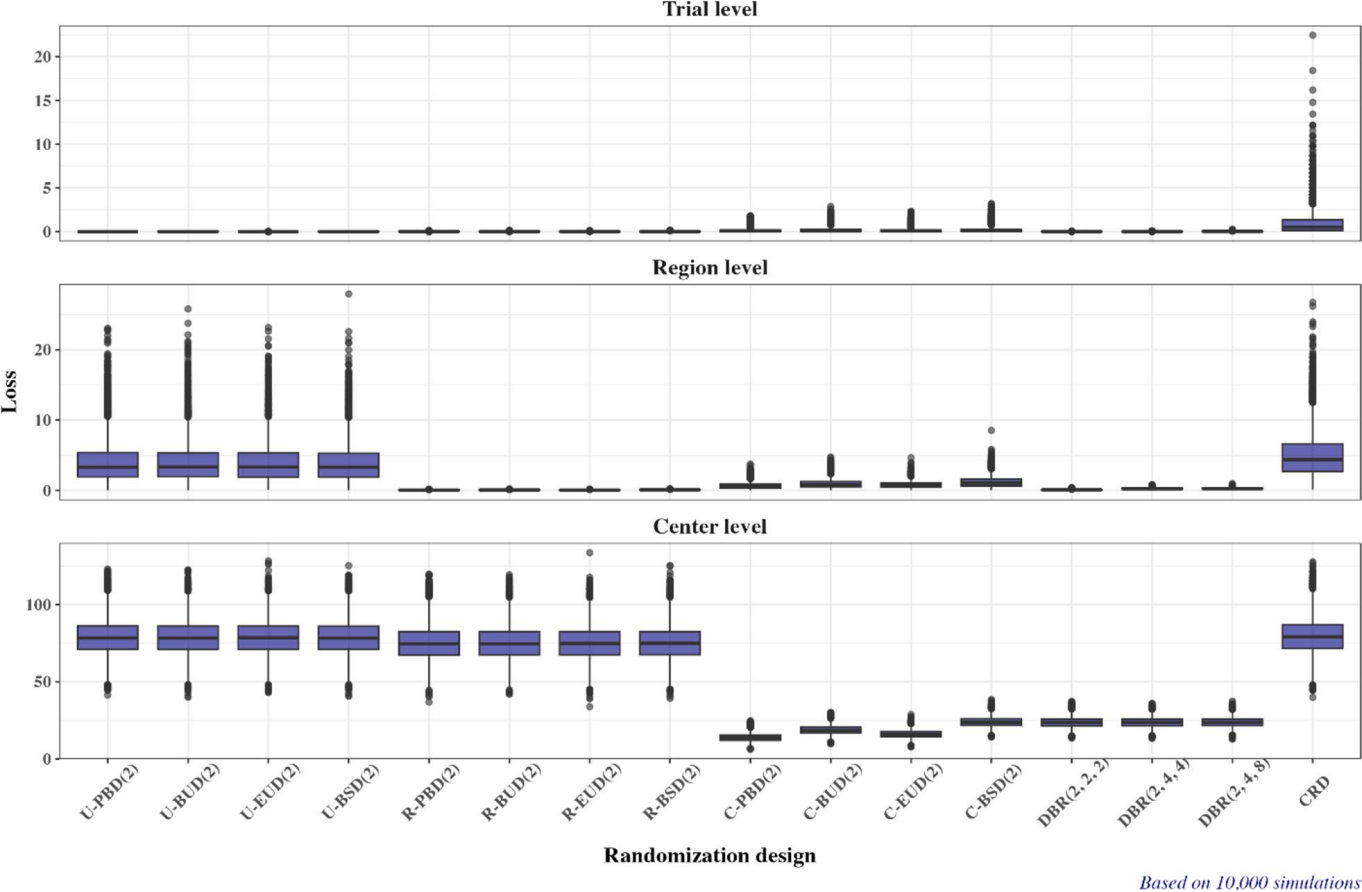
Boxplots of loss at the trial, region, and center level for 16 randomization designs under Scenario 1

Figure 3 shows the boxplots of the distributions of loss at the trial, region, and center level for 16 randomization designs under Scenario 1. The following important observations can be made:

- The four unstratified restricted randomization procedures—U-PBD(2), U-BUD(2), U-EUD(2), and U-BSD(2)—result in the lowest loss at the trial level but they fail to control imbalance at the region and center levels.
- The four region-stratified randomization procedures—R-PBD(2), R-BUD(2), R-EUD(2), and R-BSD(2)—do a very good job keeping the loss low at both the region and the trial levels, but they are almost equivalent to CRD at the center level.
- The four center-stratified randomization procedures—C-PBD(2), C-BUD(2), C-EUD(2), and C-BSD(2)—have a pretty good control of loss at all three levels. They tend to have the lowest lost among all considered designs at the center level, and they maintain reasonably small values of loss (way below those of CRD) at both the region and the trial levels. Among the four center-stratified designs, the loss tends to be lowest for C-PBD(2) and highest for C-BSD.
- The three dynamic balancing randomization procedures—DBR(2, 2, 2), DBR(2, 4, 4), and DBR(2, 4, 8)— tend to have very low loss at both the trial and the region levels, and they are similar to C-BSD(2) at the center level. There seems to be no added value due to using lower imbalance tolerance thresholds at the region and trial levels—the configurations (2, 2, 2), (2, 4, 4), and (2, 4, 8) led to almost the same distributions of loss for the DBR procedures.
- CRD tends to have highest values of loss at any level; however, the randomization procedures that do not stratify by center have similar loss to CRD at the center level; the unstratified randomization procedures have similar loss to CRD at the region level.

**Fig. 4:**
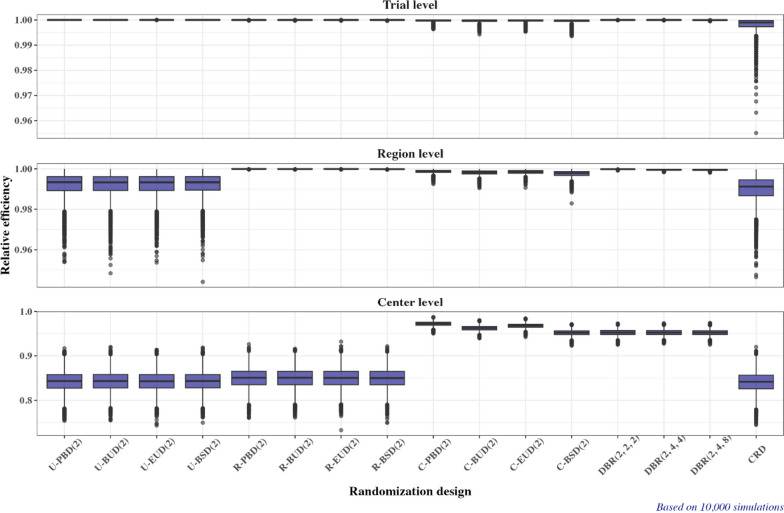
Boxplots of efficiency (relative to the idealized balanced design) in estimating the treatment effect assuming a linear model without covariates (Trial level, ${RE}_{1}$), a linear model with region as a covariate (Region level, ${RE}_{2}$), and a linear model with center as a covariate (center level, ${RE}_{3}$) for 16 randomization designs under Scenario 1

Figure 4 corroborates the findings in Fig. 3. What is important to notice is the magnitude of loss in efficiency due to imbalance induced by different randomization procedures. If responses follow a normal linear model with no covariates other than the treatment group (Fig. 4, top plot), all procedures except for complete randomization are guaranteed to be at least 99% as efficient as the perfectly balanced design (250:250 allocation). For the CRD, the minimum efficiency falls short below 96%.

If both treatment and region are important covariates (Fig. 4, middle plot), then the four region-stratified randomization designs and the three DBR procedures have the same efficiency as the “idealized” balanced design (IBD), while the four center-stratified randomization designs are at least 98% as efficient as IBD. By contrast, for the four unstratified designs and the CRD the efficiency can be as low as ~ 95%.

Finally, if both treatment and center are important covariates (Fig. 4, bottom plot), then the four center-stratified randomization designs as the three DBR procedures have median (minimum) efficiency $\ge$ 95% ($\ge$ 92%). By contrast, the four unstratified randomization designs, the four region-stratified randomization designs, and the CRD in this case have median (minimum) efficiency of ~ 85% (~ 75%).

Table 2 shows the simulated standard deviation (SD) of absolute overall imbalance for 16 randomization designs. Under Scenario 1, the four unstratified designs have the SD of at most 1.0, the four region-stratified designs have SD in the range 1.4–1.8, and the four center-stratified designs have SD in the range 5.0–6.8. For the DBR procedures, SD depends on the choice of the MTI thresholds—SD can be as low as 1.1 for DBR(2, 2, 2)—which is similar to U-BSD(2), and SD = 2.3 for DBR(2, 4, 8). The CRD is most variable with SD = 13.4.

**Table 2:** Simulated standard deviation of absolute overall imbalance $SD\left|D(n)\right|$ for 16 randomization designs under Scenarios 1–4

Randomization design	Scenario 1	Scenario 2	Scenario 3	Scenario 4
U-PBD $(b)$	0.00	0.00	0.00	1.10
U-BUD $(b)$	0.95	0.94	0.94	1.14
U-EUD $(b)$	0.86	0.85	0.86	1.05
U-BSD $(b)$	1.00	1.00	1.00	1.40
R-PBD $(b)$	1.40	1.39	1.38	1.80
R-BUD $(b)$	1.57	1.58	1.58	2.30
R-EUD $(b)$	1.49	1.48	1.49	2.00
R-BSD $(b)$	1.76	1.76	1.72	3.19
C-PBD $(b)$	4.97	4.83	6.56	6.41
C-BUD $(b)$	5.93	5.46	7.29	7.58
C-EUD $(b)$	5.41	5.13	6.62	6.62
C-BSD $(b)$	6.76	6.16	8.09	10.06
DBR($b, b, b$)	1.07	1.06	1.06	1.42
DBR($b,2b,2b$)	1.45	1.43	1.45	2.41
DBR($b,2b,4b$)	2.32	2.29	2.35	4.34
CRD	13.43	13.37	13.54	13.47

Table 3 displays the simulated ${P}_{skewed}$ for 16 randomization designs. Under Scenario 1, approximately one-third (33–35%) of the centers result in an allocation more skewed than 2:1 or 1:2 following the four unstratified designs, four region-stratified designs, and CRD. The corresponding numbers are much lower—from 1.5% to 8.5%—for the four center-stratified designs and DBR.

**Table 3:** Simulated average proportion of centers (among all centers that recruited at least 2 patients) that resulted in the allocation ratio more skewed that 1:2 or 2:1 under Scenarios 1–4

Randomization design	Scenario 1	Scenario 2	Scenario 3	Scenario 4
U-PBD $(b)$	0.347	0.322	0.382	0.323
U-BUD $(b)$	0.347	0.323	0.382	0.323
U-EUD $(b)$	0.347	0.323	0.383	0.323
U-BSD $(b)$	0.347	0.322	0.381	0.324
R-PBD $(b)$	0.334	0.308	0.370	0.308
R-BUD $(b)$	0.333	0.308	0.371	0.308
R-EUD $(b)$	0.334	0.308	0.371	0.307
R-BSD $(b)$	0.335	0.308	0.371	0.310
C-PBD $(b)$	0.015	0.045	0.083	0.139
C-BUD $(b)$	0.057	0.080	0.130	0.167
C-EUD $(b)$	0.043	0.060	0.097	0.129
C-BSD $(b)$	0.085	0.119	0.195	0.265
DBR ($b, b, b$)	0.083	0.116	0.191	0.255
DBR ($b,2b,2b$)	0.083	0.117	0.191	0.259
DBR ($b,2b,4b$)	0.084	0.117	0.192	0.259
CRD	0.349	0.326	0.385	0.328

**Fig. 5:**
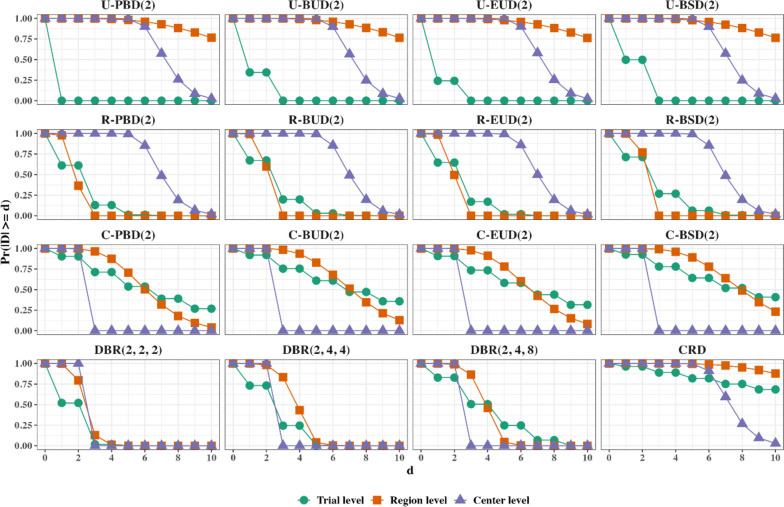
${\text{Pr}}(|Imbalance|\ge d)$ at the trial, region, and center level for 16 randomization designs under Scenario 1

Figure 5 shows the plots of ${\text{Pr}}(\left|Imbalance\right|\ge d)$ at the trial, region, and center level for 16 considered randomization designs under Scenario 1. The numerical values of these probabilities are available in Supplemental Appendix 1. The following important observations can be made:

- The four unstratified randomization procedures ensure that imbalance at the trial level (green pattern) does not exceed the MTI value (which is set to 2 in our case). However, at the region (red pattern) and the center (purple pattern) levels larger imbalances are likely—e.g., at these levels ${\text{Pr}}\left(\left|Imbalance\right|\ge 6\right)\approx 0.96$ for all 4 unstratified randomization procedures, which is similar to CRD.
- The four region-stratified randomization procedures ensure that $\left|Imbalance\right|\le 2$ at the region level. These procedures also provide a very good control of imbalance at the trial level—e.g., ${\text{Pr}}\left(\left|Imbalance\right|\ge 6\right)$ is between 1% (for R-PBD(2)) and 6% (for R-BSD(2)). However, at the center level larger imbalances are likely—e. g., ${\text{Pr}}\left(\left|Imbalance\right|\ge 6\right)\approx 0.94$ for all 4 region-stratified randomization procedures, which is similar to CRD.
- The four center-stratified randomization procedures ensure that $\left|Imbalance\right|\le 2$ at the center level. At two other levels, larger imbalances are likely; however, probability of such imbalances is smaller than for CRD. For instance, at the trial level, ${\text{Pr}}\left(\left|Imbalance\right|\ge 6\right)$ is in the range 54% to 64% for center-stratified randomization procedures whereas it is 82% for CRD. At the region level, ${\text{Pr}}\left(\left|Imbalance\right|\ge 6\right)$ is in the range 50% to 78% for center-stratified randomization procedures whereas it is 99% for CRD.
- The three dynamic balancing randomization procedures ensure best overall control of imbalance at all three levels. By design, these procedures maintain $\left|Imbalance\right|\le 2$ at the center level. At the trial level, ${\text{Pr}}\left(\left|Imbalance\right|\ge 6\right)$ is less than 1% for DBR(2, 2, 2) and DBR(2, 4, 4), and it is ~ 25% for DBR(2, 4, 8). At the region level, the corresponding values of ${\text{Pr}}\left(\left|Imbalance\right|\ge 6\right)$ are all below 1%.

**Fig. 6:**
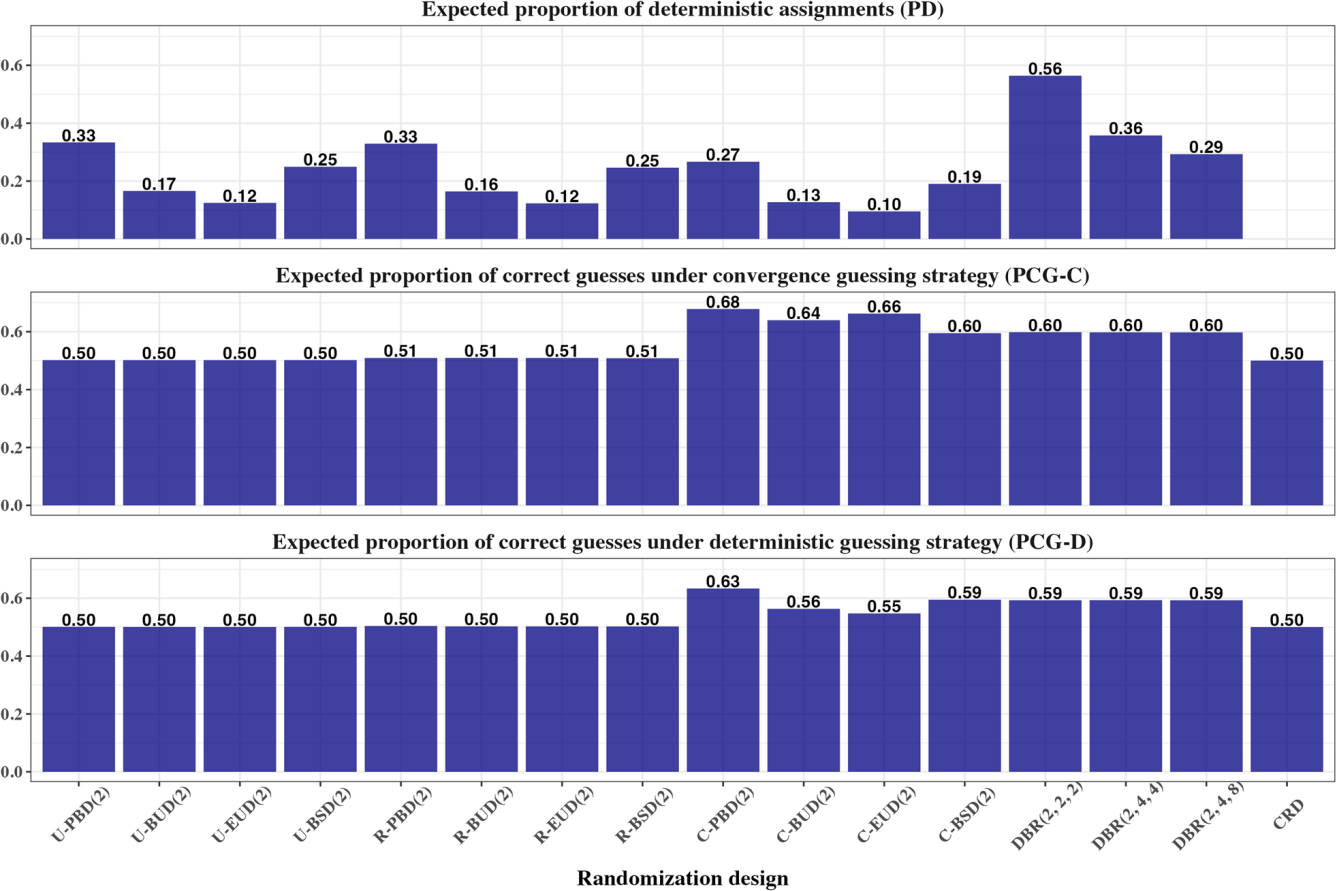
Measures of lack of randomness – expected proportion of deterministic assignments ($PD$) (upper plot), expected proportion of correct guesses under the convergence strategy ($PC{G}_{c}$) (middle plot), and expected proportion of correct guesses under the deterministic strategy ($PC{G}_{d}$) (bottom plot) for 16 randomization designs under Scenario 1

Figure 6 shows the lack of randomness measures for 16 considered randomization designs under Scenario 1. One can see that the expected proportion of deterministic assignments ($PD$) (Fig. 6, top plot) is highest for DBR(2, 2, 2) ($PD=0.56$), followed by DBR(2, 4, 4) (0.36), two permuted block randomization procedures: U-PBD(2) (0.33) and R-PBD(2) (0.33), and DBR(2, 4, 8) (0.29). The lowest $PD$ is for three procedures involving Ehrenfest urn design—U-EUD(2), R-EUD(2), and C-EUD(2)—their values of $PD$ are equal to 0.12, 0.12, and 0.10, respectively. For CRD, $PD=0$.

The expected proportion of correct guesses under convergence guessing strategy ($PC{G}_{c}$) (Fig. 6, middle plot) is largest for C-PBD(2) ($PC{G}_{c}=0.68$). By replacing permuted blocks within strata with some less restrictive MTI procedure one can lower the probability of correct guesses: for C-EUD(2), C-BUD(2), and C-BSD(2) the values of $PC{G}_{c}$ are 0.66, 0.64, and 0.60, respectively. The three DBR procedures have $PC{G}_{c}=0.60$. The four unstratified and the four region-stratified randomization procedures are similar to CRD for which $PC{G}_{c}=0.50$.

The expected proportion of correct guesses under deterministic guessing strategy ($PC{G}_{d}$) (Fig. 6, bottom plot) is, in general, not the same as $PC{G}_{c}$. The $PC{G}_{d}$ is largest for C-PBD(2) ($PC{G}_{d}=0.63$). For C-BUD(2), C-EUD(2), and C-BSD(2) the values of $PC{G}_{d}$ are 0.56, 0.55, and 0.59, respectively. For the three DBR procedures we have $PC{G}_{d}=0.59$. For all other designs (unstratified, region-stratified, and CRD), $PC{G}_{d}=0.50$.

### Scenario 2

Under Scenario 2, we have increased variability in the center recruitment rates while keeping all other parameters the same as in Scenario 1. From Fig. 1 (middle plot), the time to complete recruitment has a symmetric, bell-shaped distribution around 365 days, but it is more dispersed compared to Scenario 1—the IQR if from 344 to 391 days, and the range is 259–573 days.

Figure 2 (middle plot) shows the average number of centers that recruited exactly $j=\mathrm{0,1},\dots$ patients under Scenario 2. This plot exhibits a right-skewed distribution. On average, 9 centers would not recruit a single patient, ~ 9 centers would recruit exactly 1 patient, etc. Also, less than one site, on average, would recruit exactly 17, 18 (or more) patients.

**Fig. 7:**
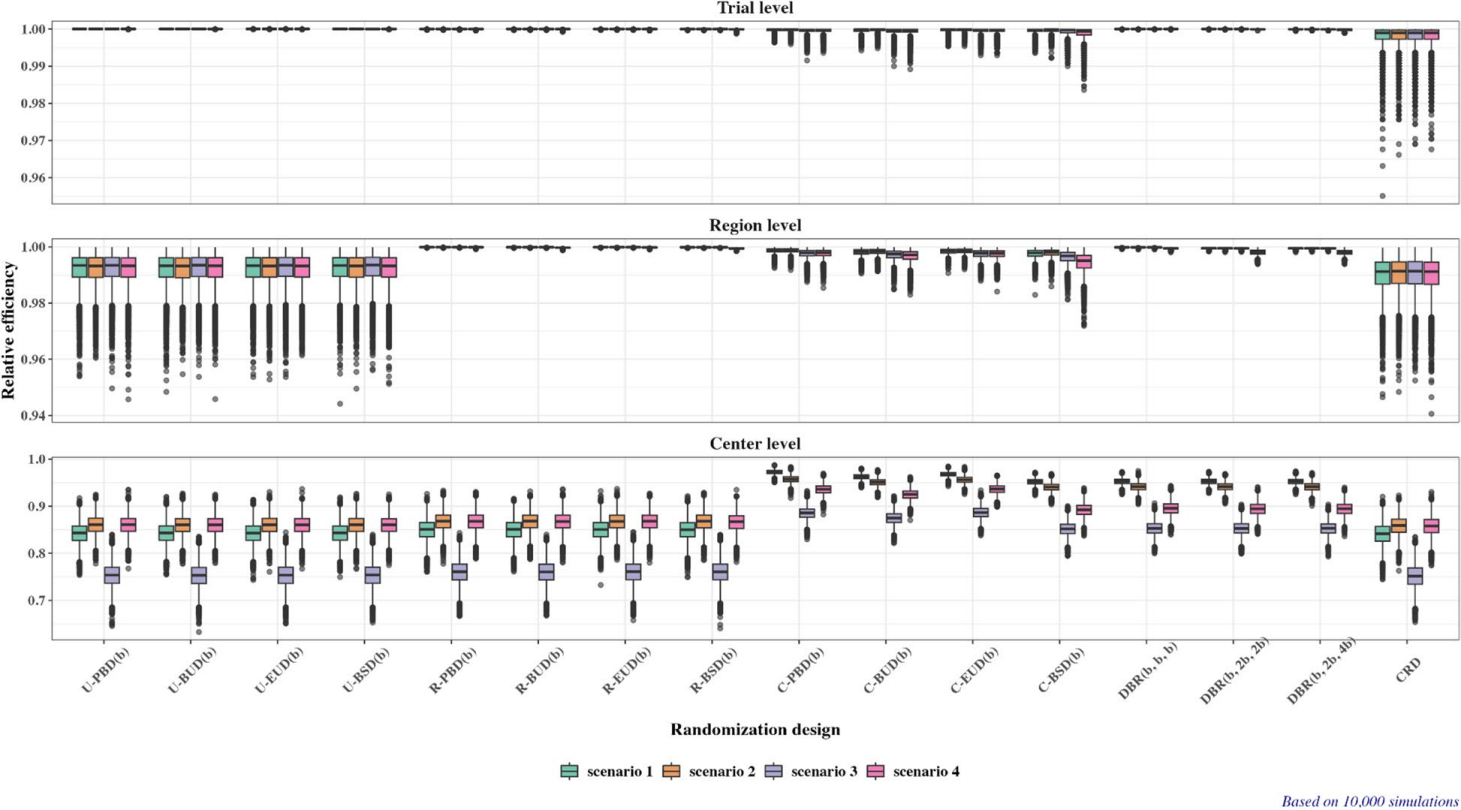
Boxplots of estimation efficiency (relative to the idealized balanced design) for 16 randomization designs under Scenario 1 (green boxplots), Scenario 2 (orange boxplots), Scenario 3 (purple boxplots), and Scenario 4 (pink boxplots)

**Fig. 8:**
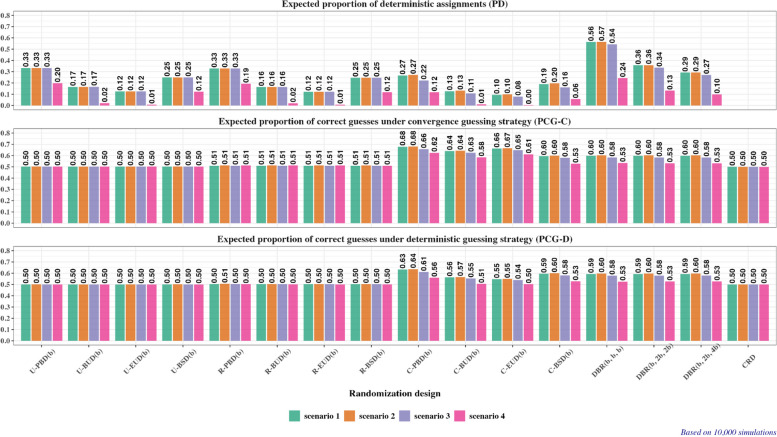
Measures of lack of randomness for 16 randomization designs under Scenario 1 (green bar plots), Scenario 2 (orange bar plots), Scenario 3 (purple bar plots), and Scenario 4 (pink bar plots). Upper plot – expected proportion of deterministic assignments ($PD$); middle plot – expected proportion of correct guesses under the convergence strategy ($PC{G}_{c}$); bottom plot – expected proportion of correct guesses under the deterministic strategy ($PC{G}_{d}$)

The full report of operating characteristics of 16 randomization designs under Scenario 2 is available in Supplemental Appendix 2. Here we focus on estimation efficiency relative to the “idealized” balanced design (Fig. 7, orange-colored boxplots) and the measures of lack of randomness (Fig. 8, orange-colored bar plots).

As regards estimation efficiency:

- Under the normal linear model with no covariates other than the treatment group (Fig. 7, top plot, orange-colored boxplots), or if both treatment and region are important covariates (Fig. 7, middle plot, orange-colored boxplots), all 16 randomization designs have very similar efficiency compared to Scenario 1.
- Under the normal linear model with both treatment and center as important covariates (Fig. 7, bottom plot, orange-colored boxplots), the four unstratified designs, the four region-stratified designs, and the CRD have, overall, slightly higher efficiency in Scenario 2 than in Scenario 1. By contrast, the four center-stratified designs and the three DBR procedures have, overall, slightly lower efficiency in Scenario 2 than in Scenario 1. This may be due to that in Scenario 2 we have increased number of centers that recruited very few (1–3) patients compared to Scenario 1.

As regards allocation randomness, the values of $PD$, $PC{G}_{c}$, and $PC{G}_{d}$ of 16 randomization designs under Scenario 2 (Fig. 8, orange-colored bar plots) are the same as the corresponding values under Scenario 1 (Fig. 8, green-colored bar plots).

From Table 2, the values of $SD\left|D(n)\right|$ of most randomization designs under Scenario 2 are about the same as in Scenario 1. From Table 3, the values of ${P}_{skewed}$ of the four unstratified randomization designs, four region-stratified designs, and CRD under Scenario 2 are about the same as the corresponding values in Scenario 1; however, for the four center-stratified designs and the three DBR designs, ${P}_{skewed}$ is increased by ~ 2–4 percentage points in Scenario 2 compared to Scenario 1.

Overall, increased variability of the center recruitment rates affects the operational aspects of the study (time to complete recruitment, numbers of patients recruited per center), but it seems to have little impact on balancing properties/estimation efficiency of randomization designs and no impact on the measures of randomness/predictability of randomization designs.

### Scenario 3

Under Scenario 3, the number of centers is increased from $N=80$ to $N=160$ and the variability of center recruitment rates is increased compared to Scenario 1 (it is kept the same as in Scenario 2). From Fig. 1 (right plot), the time to complete recruitment has a symmetric, bell-shaped distribution, shifted to the left from 365 days (median = 215 days; IQR is 206–224 days; and range is 172–272 days). Therefore, the recruitment is accelerated due to additional centers.

From Fig. 2 (right plot), the average number of centers that recruited exactly $j=\mathrm{0,1},\dots$ patients has a right-skewed distribution, similar to that in Scenario 2; however, now we have, on average, more centers (~ 35) that recruited no patients, ~ 30 centers that recruited exactly 1 patient, etc. Fewer than one center, on average, has recruited exactly 13, 14 (or more) patients.

The full report of operating characteristics of 16 randomization designs under Scenario 3 is available in Supplemental Appendix 2. Here we focus on estimation efficiency relative to the “idealized” balanced design (Fig. 7, purple-colored boxplots) and the measures of lack of randomness (Fig. 8, purple-colored bar plots).

As regards estimation efficiency:

- Under the normal linear model with no covariates other than the treatment group (Fig. 7, top plot, purple-colored boxplots), all 16 randomization designs have very similar efficiency compared to Scenario 1.
- If both treatment and region are important covariates (Fig. 7, middle plot, purple-colored boxplots), for 12 randomization designs (4 unstratified, 4 region-stratified, 3 DBR, and CRD) the findings are very similar to those in Scenarios 1 and 2. For the 4 center-stratified designs there is a slight drop in efficiency (~ 1%) compared to the results in Scenarios 1 and 2.
- If both treatment and center are important covariates (Fig. 7, middle plot, purple-colored boxplots), all 16 randomization designs exhibit a substantial drop in estimation efficiency compared to Scenarios 1 and 2. With the 4 center-stratified and 3 DBR procedures, the median (minimum) efficiency is now ~ 85–87% (80%). For the 4 unstratified procedures, 4 region-stratified procedures, and the CRD the corresponding values of median (minimum) efficiency are ~ 75% (65%). This represents an absolute decrease in efficiency of ~ 10% compared to Scenarios 1 and 2.

As regards allocation randomness:

- From Fig. 8 (upper plot, purple-colored bar plots), the 4 unstratified designs, 4 region-stratified designs, and CRD have the same values of $PD$ in Scenario 3 as in Scenarios 1 and 2. For the 4 center-stratified designs and the 3 DBR designs, there is an absolute decrease of 2–5% in $PD$ in Scenario 3 compared to Scenarios 1 and 2. For instance, C-PBD(2) has $PD=22\%$ in Scenario 3 vs. 27% in Scenarios 1 and 2.
- From Fig. 8 (middle and bottom plots, purple-colored bar plots), the 4 unstratified designs, 4 region-stratified designs, and CRD have the same values of $PC{G}_{c}$ and $PC{G}_{d}$ across Scenarios 1, 2, and 3. The 4 center-stratified designs and the 3 DBR designs exhibit 1–2% absolute decrease in $PC{G}_{c}$ and $PC{G}_{d}$ in Scenario 3 compared to Scenarios 1 and 2.

From Table 2, the values of $SD\left|D(n)\right|$ of 12 randomization designs (4 unstratified, 4 region-stratified, 3 DBR, and CRD) are about the same across Scenarios 1, 2, and 3; however, in the majority of cases for the four center-stratified designs, there is ~ 33–34% relative increase in $SD\left|D(n)\right|$ in Scenario 3 compared to Scenarios 1 and 2. From Table 3, the values of ${P}_{skewed}$ of the four unstratified randomization designs, four region-stratified designs, and CRD increased by ~ 3–4 percentage points in Scenario 3 compared to Scenario 1; and for the four center-stratified designs and the three DBR designs, ${P}_{skewed}$ is increased by 5–11 percentage points in Scenario 3 compared to Scenario 1.

Overall, doubling the number of centers from $N=80$ to $N=160$ helps expedite the completion of recruitment to some extent; however, it also increases the number of sites that recruited no patients or very few patients. Under Scenario 3, treatment imbalance at the trial and region level is likely to be similar to Scenarios 1 and 2 for all 16 considered randomization designs. However, at the center level, the imbalance is likely to increase for all designs, which can lead to ~ 10% absolute loss in efficiency of treatment effect estimation under a normal linear model with both treatment and center as important covariates. This makes sense because under Scenario 3 one expects many centers with very few patients that may result in unbalanced allocation. The measures of randomness/predictability of randomization designs under Scenario 3 are either the same (for unstratified, region-stratified, or CRD) or slightly lower (for center-stratified or DBR designs) compared to Scenarios 1 and 2.

### Scenario 4

Under Scenario 4, the MTI threshold is increased for all considered randomization designs, while all other parameters are kept as in Scenario 2. Neither the time to complete recruitment nor the average number of centers that recruited a given number of patients are affected by the choice of MTI; therefore, these characteristics are identical in Scenario 4 and Scenario 2 (see Fig. 1, middle plot and Fig. 2, middle plot).

The full report of operating characteristics of 16 randomization designs under Scenario 4 is available in Supplemental Appendix 2. Here we focus on estimation efficiency relative to the “idealized” balanced design (Fig. 7, pink-colored boxplots) and the measures of lack of randomness (Fig. 8, pink-colored bar plots).

As regards estimation efficiency:

- The 4 unrestricted randomization designs, 4 region-stratified randomization designs, and the CRD in Scenario 4 have about the same efficiency as in Scenario 2.
- For the 4 center-stratified designs, efficiency is somewhat degraded in Scenario 4 compared to Scenario 2. Among these designs, C-BSD($b$) exhibits the highest loss in efficiency (Fig. 7, pink-colored boxplots).
- For the 3 DBR procedures, there is also some degradation in efficiency in Scenario 4 compared to Scenario 2. For example, median (minimum) efficiency under a linear model with additive treatment and center effects (Fig. 7, bottom plot, pink colored boxplots) of the 3 DBR procedures is ~ 90% (~ 83%) in Scenario 4 vs. ~ 94% (~ 90%) in Scenario 2.

As regards allocation randomness, there is a substantial reduction in $PD$ for all considered randomization designs (except for CRD which already has $PD$ $\equiv 0$) (Fig. 8, top plot, pink-colored bar plots). For instance, randomization designs based on Ehrenfest urn or block urn have $PD$ of at most 2% under Scenario 4). For the 3 DBR procedures, a 2.3–2.9-fold decrease in $PD$ is observed under Scenario 4 compared to Scenario 2. Likewise, $PC{G}_{c}$ and $PC{G}_{d}$ of the 4 center-stratified randomization designs and the 3 DBR procedures is decreased by 5–7 percentage points compared to Scenario 2.

## Conclusions

### Summary and discussion

Multi-center randomized clinical trials are increasingly common in clinical research. The choice of a fit-for-purpose randomization method for a multi-center RCT is not straightforward. Many factors should be considered at the study planning stage, such as the sample size, the number of centers and their geographic location, the stochastic recruitment of patients, amongst others. There are plenty of methods to sequentially randomize patients in a multi-center RCT, with or without considering stratification factors. These methods vary in the degree of treatment balance and allocation randomness, as well as the type of randomization mechanism—for some procedures the randomization sequence can be pre-generated, whereas for others it can only be generated dynamically, depending on the covariates of the new trial participant and the covariates of already randomized trials participants.

In this work, we investigated four different types of randomization—unstratified, region-stratified, center-stratified, and dynamic balancing randomization. Within each type, we explored different possibilities for the choice of a randomization procedure (e.g., conventional permuted block design vs. a less restrictive MTI procedure), and for a given procedure we explored the choice of the block size(s) and MTI threshold(s). Furthermore, we investigated different metrics of treatment balance and allocation randomness. Balance in treatment assignments is frequently required at different levels—e.g., overall in the trial, across different geographic regions, across study centers, etc. A randomization design that achieves balance at a given level (e.g., trial) may be suboptimal in terms of balance at other levels (e.g., region or center). Moreover, a randomization design that forces excessive balance may result in a high number of deterministic assignments and/or assignments that could be easily predictable using some intelligent guessing strategy, which can invite selection bias in open-label studies.

We investigated one “base case” experimental scenario and three additional scenarios by varying the input parameters of the Poisson-gamma recruitment model or the randomization parameters (block size and MTI threshold). Our major findings can be summarized as follows:

1. Maximum tolerated imbalance (MTI) randomization procedures provide a very good alternative to the conventional permuted block design (PBD). The former procedures ensure the same amount of treatment balance at trial, region, and center level, while being more random and less predictable than PBD.
2. Unstratified randomization methods ensure treatment balance at the trial level, but they behave like complete randomization at the region or center levels. Region-stratified randomization methods (in our simulations we assumed 5 geographic regions) ensure balance at the region level and the trial level, but they behave like complete randomization at the center level. Center-stratified randomization methods control imbalance at the center level and provide a reasonable (but not ideal) control of imbalance at both the region and the trial levels. Dynamic balancing randomization (DBR) methods do a very good job simultaneously controlling imbalance at all 3 levels (trial, region, and center). A well-balanced experiment translates into accurate (unbiased, low variance) estimates of the treatment effect under a normal linear model for the response (and possibly other models, not explored in the current work), and is very appealing from the drug supply perspective.
3. The correct guess probability (CGP) under the Blackwell-Hodges model using the convergence strategy or using the deterministic guessing strategy applied by investigators at the study site level can be considerable for center-stratified randomization and DBR methods in an open-label trial. Stratified permuted block design has highest CGP among the considered methods. For region-stratified or unstratified randomization, the strategy of guessing treatment assignments in a sequence at the center level is futile, and so for these methods CGP is close to that of a random guess and should not be a concern.
4. An increased heterogeneity in center recruitment rates may increase uncertainty in recruitment characteristics (time to complete recruitment and numbers of patients recruited per center) but it does not affect the properties of the randomization procedures. (Note that changing the center recruitment rates may or may not be under the sponsor’s control.)
5. Adding more centers into the study (e.g., doubling the number of centers from 80 to 160 ) helps accelerate the recruitment process but at the expense of increasing the number of centers that recruited very few (or no) patients. This does not impact balancing or randomness properties of unstratified or region-stratified randomization designs; however, it increases the chance of imbalance at the trial level for center-stratified and DBR procedures. Therefore, if both treatment and center are important covariates, the efficiency of estimating the treatment effect can be decreased for these randomization methods.
6. Increasing the value of the block size or the MTI threshold(s) helps substantially improve the randomness properties of the procedures (e.g., lower the proportion of deterministic assignments and/or the proportion of correct guesses in the treatment allocation sequence), but at the expense of some extra imbalance, which may—for some procedures—translate into some loss in estimation efficiency, especially under a linear model with additive treatment and center effects. Overall, increasing the block size or the MTI threshold(s) may help obtain designs with improved randomness–balance tradeoff.
7. It is difficult to recommend any particular randomization method as the “winner”. In practice, a careful investigation of different randomization design options under standard to worst-case scenarios would be helpful. Finding an “optimal” value of MTI threshold(s) for selected design(s) can be done using Monte Carlo simulations.

In summary, our simulation evidence suggests that the choice of a randomization method impacts statistical properties of a multi-center RCT with a stochastic recruitment process, and a careful assessment of different options is warranted at the study planning stage.

### Limitations and Future Work

While our study has provided many useful insights, it has some limitations. Our considered criteria of balance were directly related to the differences in treatment assignments between the groups—overall in the trial, within-region, and within-center. It is well established that for a homoscedastic linear model with fixed additive effects of treatment and selected covariates (study center or geographic region in our examples), the most efficient design for estimating the treatment effect is one that balances treatment assignments over the distribution of the covariates [46]. Hence, center-stratified, region-stratified, and dynamic balancing randomization procedures considered in our paper were legitimate choices as they pursued statistically most efficient allocations under the fixed-effects homoscedastic linear model framework with the specified covariate structures. While we did not investigate the issue of statistical inference following the randomization designs, a proper analysis approach based on the fixed-effects linear model would adjust for all stratification variables. This is also consistent with the 2015 EMA guideline [11]:

“…In multicentre trials randomisation might be stratified by centre, country and/or region. The stratification variables used for randomisation should be adjusted for in the primary analysis.”

However, what if the treatment effect differed across study centers? What if many centers enrolled only a few participants? What if some centers randomized their enrolled participant(s) to only one treatment? Some additional important methodological challenges in this context are related to: 1) whether a statistical model should allow for the treatment-by-center interaction; and 2) whether the center should be regarded as a fixed or random effect. In this regard, the 1998 ICH E9 guideline [2] has the following paragraphs:

“The statistical model to be adopted for the estimation and testing of treatment effects should be described in the protocol. The main treatment effect may be investigated first using a model which allows for centre differences, but does not include a term for treatment-by-centre interaction. If the treatment effect is homogeneous across centres, the routine inclusion of interaction terms in the model reduces the efficiency of the test for the main effects. In the presence of true heterogeneity of treatment effects, the interpretation of the main treatment effect is controversial...”

“...Up to this point the discussion of multicentre trials has been based on the use of fixed effect models. Mixed models may also be used to explore the heterogeneity of the treatment effect. These models consider centre and treatment-by-centre effects to be random, and are especially relevant when the number of sites is large.”

In the literature, several papers investigated both fixed-effects and random-effects models of analysis of multi-center RCT data [47–51]. The conclusions and recommendations from these papers are not uniform. For instance, the authors of [49] provide some insightful simulation evidence showing that “where centre effects are small and recruitment in many centres is low, the approaches of ignoring centres or incorporating them as random effects have better performance than fixed effects analysis.” The latter findings on advantages of a random-effects model were further corroborated in the papers [50, 51]. These works suggest that it may be useful to consider the measures of imbalance and corresponding loss in statistical efficiency for a class of models that regard the center as a random effect. In this case, the variance of the treatment effect estimator is generally different from that under the fixed-effects model [29], and one may expect that the benefits of various randomization methods may be different from the ones obtained in the current paper. These issues merit further investigation and we defer it to the future work.

Our investigation considered only study center or geographic region as stratification factors. However, stratification can be also applied on selected patients’ prognostic factors observed at baseline that may be strongly related to the primary outcome. Investigating stratified randomization schemes that use a combination of administrative factors (e.g., center or region) and prognostic factors (e.g., age, sex, disease severity, etc.) may be an interesting and important research problem.

In the present paper we focused on 1:1 RCTs; however, in practice many trials use unequal target allocation ratios, e.g., 2:1, and/or may involve more than two arms, e.g., dose–response studies, platform trials, etc. The choice of a randomization method in a multi-center multi-arm RCT with possibly unequal allocation ratios in an important problem worthy investigating. Some relevant work was done in [19]; however, these authors mainly focused on permuted block randomization, whereas many other randomization methods could provide potentially better alternatives [52, 53].

In our present work we considered the Poisson-gamma model for patient recruitment [29, 30], which is well-established and widely used in multi-center clinical trials. Other models for recruitment could be considered [54]. It may be interesting to explore the robustness of our findings under recruitment models with additional level of sophistication, such as a hierarchical Poisson-gamma model [55], a Poisson–Pareto model [56], amongst others.

In our study we considered only one dynamic randomization procedure, the DBR method [23]. However, there are other randomization methods that could be useful in this context. For example, Zelen [27] proposed a randomization procedure for a multi-center 1:1 RCT based on a pre-generated central randomization schedule that allows skipping treatment assignments if center-level imbalance exceeds a pre-specified threshold. McEntegart [28] modified Zelen’s approach by introducing a mechanism to fill the gaps in the allocation schedule to ensure good balance in treatment assignments even in a small study. As demonstrated in [57], equal allocation modified Zelen's approach provides a good within-center balance in treatment assignments, operates within a limited drug stock, and provides a good across-study balance in treatment assignments even in a moderate size study. It would be interesting to explore the performance of the “modified Zelen's approach” and DBR in a head-to-head comparison.

Finally, in our current work we focused on balance and randomness (and their tradeoff) but did not investigate the statistical inference criteria such as power and type I error. The latter problem necessitates the formulation of a statistical model for the primary outcome, which can be continuous, binary, count, or time-to-event. Furthermore, one would have to specify a statistical procedure, e.g., a two-sample t-test or analysis of covariance (ANCOVA) for continuous outcomes; Fisher’s exact test or logistic regression for binary outcomes, etc. The definition of “loss” in statistical efficiency would have to be specified accordingly. Finally, the choice of the analysis method—population model-based or randomization-based inference—could be another important consideration, as the two methods may not be equivalent if the population model assumptions are violated [7].

We hope to address the aforementioned problems in the future work.

### Supplementary Information

**Additional file 1: Appendix 1.** Probability that the absolute imbalance at the trial, region and center level is at least d=0, 1, 2,… for 16 considered randomization designs under Scenarious 1-4.

** Additional file 2: Appendix 2.** Operating characteristics (summary statistics) of Loss at the trial, region and the center level for 16 considered randomization designs under Scenarious 1-4.

** Additional file 3:** **Appendix 3.** Proof of the formulas for loss.

## Data Availability

All results reported in this paper are based either on theoretical considerations or simulation evidence. The computer code (using R and Julia programming languages) is fully documented and is available upon reasonable request.
