# Supplementary material for: Selecting a randomization method for a multi-center clinical trial with stochastic recruitment considerations
Source: BMC Med Res Methodol. 2024 Feb 28;24:52. doi: 10.1186/s12874-023-02131-z (PMC10900599; doi:10.1186/s12874-023-02131-z)
Supplement: Supplementary file 3 — Additional file 3: Appendix 3. Proof of the formulas for loss. [file 12874_2023_2131_MOESM3_ESM.docx]

## Supplemental Appendix 3: Proof of the formulas for loss

We will prove Eq. (9) which corresponds to the case when study center is an important covariate.

For the linear model $\boldsymbol{Y}=\boldsymbol{Z}'\boldsymbol{\beta}+\alpha\boldsymbol{t}+\boldsymbol{\varepsilon}$, we have $\boldsymbol{Z}=\boldsymbol{Z}_{n\times N}=[\boldsymbol{1} \boldsymbol{z}_{1}\ldots\boldsymbol{z}_{N-1}]$ (assuming $N\geq2$), where $\boldsymbol{1}$ is an $n\times1$ vector of ones, $\boldsymbol{z}_{i}$ is an $n\times1$ vector that has $n_{i}$ entries equal to 1 (for those $n_{i}$ patients that have been recruited by center $i$) and the remaining $n-n_{i}$ entries equal to 0, $i=1,\ldots,N-1$. In this case

| $\boldsymbol{Z'Z=}\left( \begin{matrix} \begin{matrix} \begin{matrix} n \\ n_{1} \end{matrix} & \begin{matrix} n_{1} \\ n_{1} \end{matrix} \end{matrix} & \begin{matrix} \begin{matrix} \boldsymbol{\ldots} \\ \end{matrix} & \begin{matrix} n_{N-1} \\ 0 \end{matrix} \end{matrix} \\ \begin{matrix} \begin{matrix} \boldsymbol{\ldots} & \end{matrix} \\ \begin{matrix} n_{N-1} & 0 \end{matrix} \end{matrix} & \begin{matrix} \begin{matrix} \ldots\\ \end{matrix} & \begin{matrix} \\ n_{N-1} \end{matrix} \end{matrix} \end{matrix} \right)$ and $\boldsymbol{Z't=}\left( \begin{matrix} \begin{matrix} \sum_{i=1}^{N-1} D_{i}\left( n \right)+D_{N}(n) \\ D_{1}\left( n \right) \end{matrix} \\ \begin{matrix} \boldsymbol{\ldots} \\ D_{N-1}(n) \end{matrix} \end{matrix} \right)$, |  |
| --- | --- |

where $D_{i}\left( n \right)=\boldsymbol{t}'\boldsymbol{z}_{i}$for $i=1,\ldots,N$. Furthermore,

$$\left( \boldsymbol{Z}^{\boldsymbol{'}}\boldsymbol{Z} \right)^{-1}\boldsymbol{=}\frac{1}{n_{N}}\left( \begin{matrix} 1 & -1 & \begin{matrix} -1 & \ldots& -1 \end{matrix} \\ -1 & 1+\frac{n_{N}}{n_{1}} & \begin{matrix} 1 & \ldots& 1 \end{matrix} \\ \begin{matrix} -1 \\ \ldots\\ -1 \end{matrix} & \begin{matrix} 1 \\ \ldots\\ 1 \end{matrix} & \begin{matrix} 1+\frac{n_{N}}{n_{2}} & \ldots& 1 \\ \ldots& \ldots& \ldots\\ 1 & \ldots& 1+\frac{n_{N}}{n_{N-1}} \end{matrix} \end{matrix} \right)$$

After some direct matrix algebra calculations, it follows that $\boldsymbol{t}'\boldsymbol{Z}\left( \boldsymbol{Z}'\boldsymbol{Z} \right)^{-1}\boldsymbol{Z}'\boldsymbol{t}$ $=\sum_{i=1}^{N} \frac{\left\{ D_{i}(n) \right\}^{2}}{n_{i}}$. Exactly the same arguments can be applied to prove Eq. (8) for the case when geographic region is an important covariate.
